# E3 Ubiquitin Ligase Mind Bomb 1 is a Novel Negative Regulator of Tumor-Suppressive BMP Signaling

**DOI:** 10.1158/2767-9764.CRC-25-0760

**Published:** 2026-08-17

**Authors:** Charisa L. Cottonham, Gloria E. Hernandez, Tommy K. Cheung, Christopher M. Rose, Rajini Srinivasan, Robert Piskol, Xiaosai Yao, Christian W. Siebel

**Affiliations:** 1 Department of Discovery Oncology, Genentech, South San Francisco, California.; 2 Department of Research Oncology, Genentech, South San Francisco, California.; 3 Department of Proteomic and Genomic Technologies, Genentech, South San Francisco, California.; 4 Computational Sciences Center of Excellence, Genentech, South San Francisco, California.; 5 Department of Cell Therapy, Genentech, South San Francisco, California.

## Abstract

Mind bomb 1 (MIB1), an E3 ubiquitin ligase required for NOTCH activation, has been identified as a strongly selective cancer dependency in genome-wide loss-of-function screens in more than 1,000 cancer cell lines. However, MIB1-correlating dependencies in these screens unexpectedly linked MIB1 not to Notch but rather to the family of transforming growth factor-β and bone morphogenic protein (BMP) signaling molecules. In this study, using genetic and pharmacologic methods, we show that MIB1 loss phenocopies BMP tumor-suppressive function. MIB1-dependent cancer cell lines were sensitive to BMP ligands, and MIB1 loss selectively enhanced BMP signaling, as evidenced by SMAD1/5/9 phosphorylation and transcriptional responses that correlated with increased BMP receptor protein levels. Growth inhibition and enhanced signaling were both reversed by inhibitors of BMP type 1 receptors, demonstrating that these MIB1 effects directly reflect a function for MIB1 as a negative regulator of BMP signaling. Unlike wild-type MIB1, reexpression of RING domain–deleted MIB1 failed to rescue growth inhibition in MIB1-depleted cells, suggesting that negative regulation depends on E3 ligase activity. Supporting this regulatory role, we found that MIB1 and the BMP type II receptor BMPR2 physically associate in both cancer and endothelial cells. By revealing this previously unknown role for MIB1 as a Notch-independent negative regulator of BMP signaling, our study expands the significance of MIB1 in cancer biology, with implications for targeting BMP signaling in cancer and other diseases.

**Significance:**

This study identifies MIB1 as a Notch-independent, E3 ligase–dependent regulator of BMP signaling through control of BMPR2 protein abundance, revealing a mechanistic vulnerability in BMP-responsive cancers and a potential therapeutic target.

## Introduction

The transforming growth factor-β (TGF-β) family comprises numerous secreted factors regulating various physiologic functions, from embryonic development to immune modulation (1). While tightly regulated for tissue homeostasis, dysregulated TGF-β signaling contributes to diseases such as cancer, fibrosis, autoimmune disorders, and developmental abnormalities (2). In cancer, TGF-β signaling demonstrates a complex interplay, exhibiting both oncogenic and tumor-suppressive functions. Although it can suppress immune surveillance and promote fibrosis in the tumor microenvironment, it also impedes cell-cycle progression and induces apoptosis in transformed epithelial cells (2, 3). This dual role is context-dependent, with the tumor-suppressive function often acting directly on tumor cells during early carcinogenesis, whereas the oncogenic role is predominantly mediated by immune and stromal cells (2, 3).

The TGF-β family encompasses two distinct subfamilies: one comprising TGF-β isoforms, activins, and nodal, and the other includes bone morphogenic proteins (BMP), growth differentiation factors, and Mullerian inhibitory substance (4). Each subfamily binds to specific type I and type II serine/threonine kinase receptor pairs. TGF-β isoforms primarily bind to type I receptor TGFBR1 (ALK5) and type II receptor TGFBR2 (4). Meanwhile, BMPs primarily bind to type I receptors ACVRL1 (ALK1), Bmpr1a (ALK3), and Bmpr1b (ALK6) paired with type II receptor BMPR2 (4). These subfamilies also engage distinct receptor SMADs, with the TGF-β subfamily signaling through Smad2 and Smad3, whereas the BMP subfamily predominantly activates Smad1, Smad5, and Smad9 (also known as Smad8; ref. 4). The diversity of ligand–receptor pairs within the TGF-β family imparts a wide range of functional outcomes.

Irrespective of subfamily distinction, the signaling cascade from the cell membrane to the nucleus follows a conserved mechanism. Ligand binding to the type II receptor induces conformational changes in the receptor complex, leading to transphosphorylation of the type I receptor by the constitutively active type II receptor. The activated type I receptor phosphorylates receptor Smads, which then form complexes with Smad4 and translocate into the nucleus to regulate gene expression, thereby affecting cellular processes.

Several steps of the TGF-β family signaling cascade are subjected to both positive and negative regulation to fine-tune pathway activation and downstream effects. Ligand availability and receptor binding are modulated by various factors such as extracellular antagonists, glycoproteins, extracellular matrix proteins, and decoy receptors (5–7). Receptor endocytosis and degradation, facilitated by E3 ligases like Smurf1, further contribute to signaling modulation (5). Nuclear transport factors, including importins and exportins, regulate Smad complex translocation, ensuring proper signal transmission within the nucleus (8, 9). This intricate regulatory network guarantees precise control of TGF-β family signaling in both physiologic and pathologic contexts.

Mind bomb 1 (MIB1) is a broadly expressed multifunctional protein, best known for its essential role in Notch pathway activation. MIB1-mediated ubiquitination of Notch ligands is crucial for their internalization and subsequent receptor activation (10). Beyond Notch signaling, MIB1 ubiquitinates various substrates, including AZI1 and PCM1 (centrosomal functions), CDKL5 (neuronal motility), p120 catenin (cell junctions), GABARAP (autophagosome), and EPB41L5 (cell polarity; refs. 11–15). MIB1 is also implicated in RNA metabolism, regulation of splicing factors, and viral infection by facilitating adenovirus nuclear entry (16, 17). Despite its diverse roles, no connections between MIB1 and TGF-β family signaling have been reported.

MIB1’s versatility arises from its structural regions. The MZM–REP region is essential for recognizing Notch ligands (18, 19) in a bipartite mechanism (20). Its ankyrin repeat region mediates diverse protein–protein interactions (21). MIB1 has three RING finger domains, with RING3 critical for its E3 ubiquitin ligase function (15, 18, 22). MIB1’s E3 ligase activity varies contextually, acting as a monoubiquitinase for Notch ligands to regulate recycling (18, 23) and as a polyubiquitinase for substrates like PCM1 and Cep131 to promote destabilization (24). These features highlight MIB1’s adaptability and diverse functional repertoire.

Here, we uncover a role for MIB1 in the regulation of BMPR2 protein levels. We found that depletion of MIB1 in cancer cells leads to elevated phosphorylation and nuclear translocation of SMAD1/5/9, culminating in elevated BMP signaling and cell-cycle arrest in cancer cells. Notably, loss of MIB1 does not affect signaling of the TGF-β isoforms in these cells. Supporting a mechanistic link, we show that MIB1 and BMPR2 associate in both cancer and endothelial cells, suggesting that MIB1 may regulate BMP signaling through receptor-level control of BMPR2 abundance.

## Materials and Methods

Key reagents and resources used in this study are listed in Supplementary Table S1.

### Dependency map datamining

Cancer cell line dependency profiles were obtained from the Cancer Dependency Map (DepMap) portal (https://depmap.org/portal). The following datasets were used in this study: CRISPR DepMap Public 22Q2 + Score (25) and CRISPR DepMap Public 22Q4 + Score and Expression 22Q4 Public (25). Dependency scores for all cancer cell lines were categorized based on lineage types as defined by DepMap and used in downstream correlation analyses. Spearman’s correlation coefficients and *P* values derived from linear regression were obtained from precomputed associations available on the DepMap portal (26). Publicly available DepMap data were filtered and grouped by cancer lineage using basic Python 3 scripting in Google Colab (RRID: SCR_008394). Code used for processing was minimal and is available upon request.

### Cell culture

All parental human cancer cell lines were sourced from the Genentech cell bank (gCell) where they were authenticated by short tandem repeat profiling. All cell stocks were tested for *Mycoplasma* before and after cryopreservation using both the MycoAlert (Lonza) and MycoSensor (Stratagene) assays to minimize false-positive and false-negative results. All cell lines tested negative for *Mycoplasma*. BT549 (RRID: CVCL_1092, female), CAMA1 (RRID: CVCL_1115, female), HCC515 (RRID: CVCL_5136, male), HCC1187 (RRID: CVCL_1247, female), HCC1419 (RRID: CVCL_1251, female), NCIH28 (RRID: CVCL_1555, male), NCIH292 (RRID: CVCL_0455, female), and NCIH838 (RRID: CVCL_1594, male) were maintained in RPMI 1640 with 10% heat-inactivated fetal bovine serum (FBS, provided by gCell) and 2 mmol/L L-glutamine. HEK293T cells (RRID: CVCL_0V13) were maintained in DMEM with 10% heat-inactivated FBS and 2 mmol/L L-glutamine. Human umbilical vein endothelial cells (HUVEC) were purchased from Lonza (#C2519A). HUVECs were grown on gelatin-coated, tissue culture–treated six-well plates (Corning, #3516) in MCDB-131 medium (Gibco, #10372019) with 10% FBS and 2 mmol/L L-glutamine and used between passages 3 and 9. Each lot was derived from pooled donors of mixed sex. All cells were cultured in a humidified incubator at 37°C with 5% CO_2_.

### TGF-β2 purification

Human pro–TGF-β2 was expressed in stable Chinese hamster ovary cells provided by gCell. The culture supernatant containing TGF-β2 was purified using a preparative Ni-NTA affinity column. To isolate mature TGF-β2 from its latency-associated protein, subsequent purification steps were performed under denaturing conditions. The Ni-NTA elution was further processed by cation exchange chromatography on an SP HP column with a 0 to 1 mol/L NaCl gradient in 50 mmol/L MES, 20 mmol/L glycine, and 6 mol/L urea, pH 6.0, followed by reversed-phase high-performance liquid chromatography (HPLC) on a Vydac C4 column using a 5% to 95% acetonitrile gradient containing 0.1% trifluoroacetic acid (TFA). Fractions containing mature TGF-β2 were pooled and dialyzed against 1 mmol/L HCl. Protein purity (>95%) was confirmed by SDS–PAGE, and identity was validated by mass spectrometry (Agilent 6230 TOF LC/MS). This purified TGF-β2 is referred to as “GNE, PUR171061.”

### Ligand cocktails

TGF-β family ligands were combined in RPMI 1640 without supplements. Cells were treated with ligand cocktails at a final concentration of 100 ng/mL. The BMP cocktail consisted of BMP2 (Gibco, #PHC7145), BMP4 (Gibco, #PHC9534), BMP7 (Gibco, #PHC9544), and BMP9 (Novus, #3209-BP-010/CF), each at 25 ng/mL. The TGF-β cocktail consisted of TGF-β1 (Gibco, #PHG9214), TGF-β2 (GNE, PUR171061), and TGF-β3 (PeproTech, #AF-100-36E), each at 33 ng/mL.

### Small-molecule inhibitors

The following compounds were used in this study: chloroquine (CLQ, Tocris, #4109), Compound-E (Stem Cell Technologies, #73952), LDN-193189 (LDN; Tocris, #6053), SB-431542 (Tocris, #1614), and MG132 (Sigma, #474790). All inhibitors were reconstituted according to the manufacturer’s instructions and used at concentrations specified in the figure legends.

### Cell viability assay

Cells were seeded in 96-well plates and treated as indicated. After 7 to 14 days, CellTiter-Glo reagent (Promega, #G7571) was added to each well and processed according to manufacturer’s instructions. The luminescence signal was read after 30 minutes with an EnVision microplate reader (PerkinElmer).

### Clonogenic cell proliferation assay

Cells were seeded into 24-well or six-well plates at 200 to 1,000 cells per well and treated as indicated. The plated cells were allowed to grow and proliferate under normal culture conditions for 7 to 14 days, after which they were stained with crystal violet (Sigma, #HT901). Images were acquired using a flatbed scanner (HP Scanjet G4050, 1200 DPI).

### Live-cell imaging proliferation assay

Cells were seeded into 24-well or 96-well clear-bottom tissue culture plates and treated as indicated. Plates were placed into the IncuCyte S3 Live-Cell Analysis System (Sartorius, RRID: SCR_023147) for real-time imaging, with the whole well imaged under 4× magnification every 4 or 12 hours for a total of 6 days. Data were analyzed using the IncuCyte software (versions 2018A GUI and 2020B GUI).

### Immunofluorescence

Cells were plated into a 96-well PhenoPlate (Revitty, #6055302) and treated as indicated. Cells were fixed with 4% paraformaldehyde in phosphate-buffered saline (PBS) for 10 minutes at room temperature (RT). Cells were blocked with 2% FBS and 1% BSA in PBS for 1 hour at RT and incubated with primary antibodies overnight at 4°C in a humidified chamber. Alexa Fluor–conjugated secondary antibodies were used to label primary antibodies. 4′,6-Diamidino-2-phenylindole (DAPI) was used at 0.2 μg/mL to counterstain nuclei. Antibody information is provided in the “Antibodies” section of the “Materials and Methods.” Images were acquired at 40× magnification using an Opera Phenix High-Content Screening System (PerkinElmer). Images were analyzed using Columbus software (PerkinElmer), based on running Acapella version 5.1.1.128595.

### Subcellular fractionation

Cell pellets were resuspended in Buffer A [10 mmol/L HEPES, pH 7.9; 10 mmol/L KCl; 1 mmol/L EDTA; 0.1 mmol/L EGTA; 0.2% NP-40; 10% glycerol; and Halt protease and phosphatase inhibitors (Thermo Fisher Scientific, #78430)] and incubated on ice for 20 minutes with intermittent gentle mixing. Samples were briefly centrifuged, and the supernatant containing the cytoplasmic fraction was collected. The remaining pellet was washed with Buffer A and resuspended in RIPA buffer (Thermo Fisher Scientific, #89900) to lyse nuclei followed by centrifugation to clarify the nuclear lysate. To generate whole cell lysates, cells were pelleted and lysed using RIPA buffer (Thermo Fisher Scientific, #89900) with Halt Protease and Phosphatase Inhibitor Cocktail (Thermo Fisher Scientific, #78440).

### Immunoblotting

Protein concentration was determined using the Pierce bicinchoninic acid protein assay (Thermo Fisher Scientific, #23225), and 10 to 50 μg of lysate was loaded in each lane. Proteins were separated by gradient SDS–PAGE and transferred to nitrocellulose membranes. The membranes were blocked with Tris-buffered saline (TBS)-Tween, 0.05% (TBST) buffer containing 2.5% to 5% milk for 1 hour and incubated with the primary antibody in 5% BSA/TBST overnight at 4°C. Antibody information is provided in the “Antibodies” section of the “Materials and Methods.” After three washes with TBST, the membranes were incubated with fluorescent dye–conjugated or horseradish peroxidase (HRP)-conjugated secondary antibodies in TBST containing 2.5% milk for 1 hour at RT. After three washes with TBST, the membranes were imaged using either an Odyssey CLx (LI-COR) or a Chemidoc (Bio-RAD).

### Cell-cycle analysis

Cell-cycle dynamics were evaluated using the Click-it EdU Proliferation Kit (Invitrogen, #C10419) according to the manufacturer’s instructions. In brief, cells were treated with 10 μmol/L 5-ethynyl-2′-deoxyuridine (EdU) for 1 hour at 37°C with 5% CO_2_ and then fixed with 4% paraformaldehyde in PBS. Fixed cells were stained with Click-it EdU detection reagent followed by DAPI staining to distinguish different phases of the cell cycle based on DNA content. Labeled cells were detected using a BD Symphony flow cytometer. The proportion of cells in different phases of the cell cycle was determined using FlowJo software, v10. Debris and dead cells were first excluded based on forward scatter and side scatter profiles. Subsequently, doublets were discriminated against using DAPI-A and DAPI-H profiles. These singlets were then analyzed for the intensities of incorporated EdU Alexa Fluor 647 and DAPI staining. EdU-positive cells were classified as in S-phase, whereas EdU-negative cells were classified as either in G0/G1 phase or G2/M phase based on their DNA content.

### siRNA transfection

For transient knockdown, the following predesigned dicer-substrate siRNAs were used: MIB1 siRNA (IDT, hs.Ri.MIB1.13), HPRT (IDT, positive control; #51-01-08002), or a nontargeting control (IDT, #51-01-14-04). Cancer cell lines were reverse transfected with 100 nmol/L of siRNA using Lipofectamine 2000 (Thermo Fisher Scientific, #11668019) according to the manufacturer’s guidelines. HUVECs were transfected with siRNA in Buffer T using the Neon Electroporation system (Thermo Fisher Scientific, #MPK10025) according to the manufacturer’s guidelines. Forty-eight to 72 hours after transfection, protein lysates were collected using RIPA buffer (Thermo Fisher Scientific, #89900). RNA was isolated 72 hours after transfection using the RNeasy Plus Kit (Qiagen, #74134).

### BMP inhibitor washout assay

Cells were seeded in a growth medium containing either DMSO or 50 nmol/L LDN, with or without 0.5 μg/mL doxycycline (Dox). After 3 days, cells were dissociated with trypsin-EDTA, and equal cell numbers were replated into growth medium containing DMSO or 50 nmol/L LDN, along with 10 ng/mL BMP4. For RNA sequencing (RNA-seq), cells were treated with 10 ng/mL BMP4 for 24 hours before RNA was collected. For proteomics, cells were treated with or without BMP4 (10 ng/mL) for 45 minutes prior to protein collection.

### RNA-seq

MIB1 inducible knockout (MIB1-iKO) cells were seeded into six-well plates (1 × 10^5^ cells per well) and treated with 0.5 μg/mL Dox, 50 nmol/L DSMO, 10 ng/mL BMP4, or their combinations. Total RNA was extracted using RNeasy Micro Kit (Qiagen, #74004). Total RNA was quantified using Qubit RNA HS Assay Kit (Thermo Fisher Scientific, #Q32852), and quality was assessed using RNA ScreenTape on TapeStation 4200 (Agilent). For low-input RNA, cDNA library was generated from 2 ng of total RNA using Smart-Seq V4 Ultra Low Input RNA Kit (Takara, #634888), and 150 pg of cDNA were used to make sequencing libraries using Nextera XT DNA Sample Preparation Kit (Illumina, #FC-131-1096). For regular input RNA, the sequencing library was generated using the Truseq Stranded mRNA Kit (Illumina, #20020594) with an input of 100 to 1,000 ng of total RNA. Libraries were quantified using Qubit dsDNA HS Assay Kit (Thermo Fisher Scientific, #Q32851), and the average library size was determined using D1000 ScreenTape on TapeStation 4200 (Agilent). Libraries were pooled and sequenced on NovaSeq 6000 (Illumina) to generate 30 million single-end 50-base pair reads for each sample.

### RNA-seq analysis

Reads were first aligned to ribosomal RNA sequences to remove ribosomal reads. The remaining reads were aligned to the human reference genome (NCBI Assembly GRCh38) using GSNAP (27) version “October 10, 2013,” allowing a maximum of two mismatches per 50 base pair sequence (parameters: ‘-M 2 -n 10 -B 2 -i 1 -N 1 -w 200000 -E 1 --pairmax-rna=200000 --clip-overlap’). Transcript annotation was based on the human RefSeq database NCBI Annotation Releases 106. To quantify gene expression, the number of reads mapped to the exons of each RefSeq gene was calculated using the HTSeqGenie R package (RRID: SCR_005514). Read counts were scaled by library size, quantile-normalized, and precision weights calculated using the “voom” R package (28). Subsequently, differential expression analysis on the normalized count data was performed using the “limma” R package (29) by contrasting MIB1 KO samples with control samples, respectively. Gene expression was considered significantly different across groups if we observed an |log_2_ fold change (FC)| ≥ 1 (estimated from the model coefficients) associated with an FDR adjusted *P* value ≤ 0.05. In addition, gene expression was obtained in the form of normalized reads per kilobase gene model per million total reads as described previously (30).

Gene set enrichment analysis (GSEA; ref. 31) was performed on RNA-seq data from NCIH838 cells using GSEA software (RRID: SCR_003199) and the Hallmark gene sets from MSigDB (RRID: SCR_016863). Gene-level expression changes were ranked by log FC and queried using GSEA() function in clusterProfiler (RRID: SCR_016884; refs. 32, 33). Pathways with adjusted *P* values < 0.05 were considered significant.

### Construct design

All plasmid constructs were synthesized by GenScript.

#### iKO constructs

We utilized an all-in-one, Dox-inducible KO system based on a piggyBac transposon strategy that integrates a single-guide RNA (sgRNA), Cas9, and a puromycin-resistant gene. Cas9 expression is driven by Dox addition through a reverse tetracycline-controlled transactivator system. Specifically, pBIND-Cas9-sgRNA-*MIB1* and pBIND-Cas9-sgRNA-*FKBP1A* constructs were used to target MIB1 and FKBP1A, respectively. The sgRNA sequences used were as follows:

- *MIB1* sgRNA #1, 5′-TTG​GCG​CTC​GGG​TAG​TGC​G-3′.
- *MIB1* sgRNA #2, 5′-GCC​AAC​TAC​CGC​TGC​TCC​G-3′.
- *FKBP1A* sgRNA, 5′-CAC​TAC​TCA​CCG​TCT​CCT-3′.

#### MIB1 cDNA constructs

To generate a Cas9-resistant *MIB1* cDNA, a synonymous mutation was introduced into the PAM sequence targeted by *MIB1* sgRNA #2 using site-directed mutagenesis. This modification prevents cleavage by Cas9, enabling stable expression in KO backgrounds. The Cas9-resistant *MIB1* cDNA [MIB1 wild-type (WT) cDNA; based on transcript ENSG00000101752] was cloned downstream of an EF-1α core promoter in a lentiviral vector containing an IRES-linked mNeonGreen (mNG) selectable marker. The MIB1 RING domain mutant (MIB1 RING Δ1–3) was generated by deleting the sequence corresponding to amino acids 819 to 1,006 from the WT construct and cloned into the same lentiviral backbone. A lentiviral construct expressing MIB1 WT cDNA fused to mNG (MIB1-mNG) was also generated. A lentiviral construct expressing only mNeonGreen was used as a control.

#### dTAG constructs

To enable conditional degradation of exogenously expressed MIB1, a FKBP12^F36V^ degron tag was fused to the N-terminus of the Cas9-resistant MIB1 cDNA and cloned into in a piggyBac vector under the control of an EF-1α core promoter. The expression cassette also included an N-terminal 1X-Flag epitope and an IRES-linked mNeonGreen marker for selection. A piggyBac construct expressing only mNeonGreen was used as a control.

### Cell line engineering

#### iKO cells

Parental NCIH838 cells were cotransfected with a pBIND vector and a codon-optimized piggyBac transposase at a ratio of 3:1 using Lipofectamine 2000 (Thermo Fisher Scientific, #11668027), following the manufacturer’s protocol. Transfected cells were selected with 1 μg/mL puromycin for 1 week. For *MIB1*, single-cell clones were isolated by dilution cloning following transfection. In contrast, *FKBP1A*-targeted cells were maintained as a pooled population without clonal selection. Transfected cells were treated with 0.5 μg/mL Dox to induce Cas9 expression, and successful KO was confirmed by immunoblotting (IB) after 3 days. The resulting genetically modified cell lines are referred to as MIB1-iKO and FKBP1A-iKO cells.

#### MIB1-restored cells

MIB1-iKO cells were transduced with lentiviral particles generated by cotransfecting the MIB1 cDNA expression construct, packaging plasmid (delta 8.9), and a VSV-G envelope plasmid into HEK293T cells using Lipofectamine 2000. The medium was replaced 18 hours after transfection, and the virus-containing medium was harvested after 24 to 48 hours. Transduced cells were enriched by flow cytometry for the mNeonGreen-positive population and expanded. Viral clearance was confirmed using a p24 ELISA (Takara, #632200).

#### dTAG-MIB1 cells

MIB1-iKO cells were reverse transfected with dTAG constructs and a piggyBac transposon using Lipofectamine 2000. Transfected cells were sorted by flow cytometry for mNeonGreen-positive populations and expanded. Cells were then treated with Dox for 3 days to induce KO of endogenous MIB1, and MIB1 protein levels were evaluated by IB. After 2 weeks of continuous Dox treatment, Dox was withdrawn. The resulting cells are referred to as “dTAG-MIB1 cells.”

### Immunoprecipitation

Cells were lysed in Pierce IP Lysis Buffer (Thermo Fisher Scientific, #87787), and 10% to 15% of the total lysate was reserved as the input fraction. Lysates were incubated overnight at 4°C with anti-BMPR2 antibody (BD Biosciences, #612292) or isotype-matched control IgG. Pierce Protein G Magnetic Beads (Thermo Fisher Scientific, #88847) were washed with 1X IP High Buffer (Active Motif, #37510) and added to the lysate–antibody mixture for 2 hours at 4°C. Immunoprecipitates were collected using a magnetic stand, washed with 1X IP High Buffer, and eluted at 70°C for 10 minutes in NuPAGE LDS Reducing Buffer (Thermo Fisher Scientific, #NP0007 and #NP0009). Samples were analyzed by IB.

### Multiplexed quantitative proteomics sample preparation

Protein lysates (20 mg) in denaturing buffer (8 mol/L urea and 20 mmol/L HEPES, pH 8.0) were reduced [5 mmol/L dithiothreitol (DTT), 45 minutes, 37°C], alkylated (15 mmol/L iodoacetamide, 20 minutes, RT, in the dark), and quenched (5 mmol/L DTT, 15 minutes, RT, in the dark), followed by chloroform–methanol precipitation. Pellets were resuspended in denaturing buffer, diluted to 4 mol/L urea, and digested with lysyl-endopeptidase (Wako; 1:10 enzyme:protein, 4 hours, 37°C) and then diluted to 1.3 mol/L urea and digested overnight with trypsin (Promega, enzyme:protein ratio = 1:50). Peptides were acidified (1% final TFA), clarified (18,000 × g, 15 minutes), desalted on Sep-Pak C18 columns (Waters), and lyophilized. Approximately 500 μg of eluted peptides from each treatment group was lyophilized and reserved for global proteome abundance, whereas ∼10 mg of eluted peptides was used for phosphorylation analysis.

For global proteome samples, 100 μg of peptides from each sample was dissolved in 100 mmol/L HEPES, pH 8.0 (1 mg/mL). Isobaric labeling was performed using TMTPro16-plex reagents (Thermo Fisher Scientific). Each unit (0.5 mg) of TMT reagent was allowed to reach RT immediately before use. TMTPro16-plex reagents (Thermo Fisher Scientific) were reconstituted in anhydrous acetonitrile (ACN, 20 μL), added to peptides (final ACN 18%), and incubated 1 hour at RT. Reactions were quenched with 5% hydroxylamine (20 μL, 15 minutes). Labeled peptides (for global analysis) were combined in equimolar ratios, dried, redissolved in 0.1% TFA, centrifuged (16,000 × *g*), and processed for high-pH reversed-phase fractionation on an 1100 HPLC system (Agilent) using an ammonium formate-based buffer system. Peptides (400 μg) were separated over a 75-minute gradient on a Zorbax 300 Extend-C18 column (Agilent, 2.1 × 150 mm 3.5 μm), collected into 96 fractions, concatenated into 24 fractions, dried, and desalted using C18 stage tips, as previously described (34). Peptides were lyophilized and resuspended in 10 μL Buffer A [2% ACN and 0.1% formic acid (FA)] for LC-MS/MS analysis.

For phosphorylome quantitation, 10 mg of lyophilized peptides were reconstituted in 21% lactic acid (GL-Sciences, #5010-21292), 60% ACN, and 0.3% TFA. Titansphere TiO_2_ cartridges (GL Sciences, cat. #5010-21291) were sequentially equilibrated with 0.4% TFA in 80% ACN, followed by 21% lactic acid, 60% ACN, and 0.3% TFA, with each step followed by centrifugation at 100 × *g* for 1 minute. Cartridges were loaded with reconstituted samples, washed three times with 0.4% TFA and 80% ACN. Phospho-enriched peptides were eluted twice with 5% ammonium hydroxide, followed by a final elution with 60% ACN. Combined eluates were acidified with 20% TFA and dried, and isobaric labeling was performed using TMTPro16-plex reagents as above. Labeled peptides (for phospho analysis) were combined in equimolar ratios, dried, desalted with using a Sep-Pak C18 column (Waters), and reconstituted with IAP buffer without detergent. Phosphotyrosine (pY) peptides were enriched using pY PTMScan immunoaffinity beads (Cell Signaling Technology, #8803) at 4°C for 30 minutes with rotation. After centrifugation (1,500 × *g* for 30 seconds), the supernatant was retained for phosphoserine and phosphothreonine (pST) analysis. The beads were washed twice with IAP buffer, followed by three washes with water. pY-enriched peptides were eluted twice with 0.15% TFA, lyophilized, and resuspended in 2% ACN and 0.1% FA LC-MS/MS analysis.

The pST sample was cleaned with a Sep-Pak C18 column (Waters) and fractionated by high-pH reversed-phase HPLC on the same Agilent system. Peptides were separated into 96 fractions, concatenated into 12, dried, desalted, and reconstituted in Buffer A (2% ACN and 0.1% FA) for LC-MS/MS analysis.

### Mass spectrometry instrumental analysis

For global proteome and phosphorylome quantitation peptides, LC-MS/MS analysis was performed by injecting 1 μL (proteome) or 2.5 μL (phospho) of each fraction on an Orbitrap Eclipse mass spectrometer (Thermo Fisher Scientific) coupled to a Dionex Ultimate 3000 RSLC (Thermo Fisher Scientific) using a 25-cm IonOpticks Aurora Series column (IonOpticks) with a gradient of 2% to 30% Buffer B (98% ACN and 2% H_2_O with 0.1% FA, flow rate = 300 nL/minute). All samples were analyzed with a total run time of 180 minutes. The Orbitrap Eclipse collected FTMS1 scans at 120,000 resolution with an AGC target of 1 × 10^6^ and a maximum injection time of 50 milliseconds. FTMS2 scans on precursors with charge states of 3 to 6 were collected at 15,000 resolution with Collision-induced dissociation (CID) fragmentation at a normalized collision energy of 35%, an AGC target of 2 × 10^4^ (proteome) or 2 × 10^5^ (phospho), a maximum injection time of 100 milliseconds (proteome) or 200 milliseconds (phospho), and multistage activation with 97.97 neutral loss mass (phospho). Synchronous precursor selection MS3 scans were analyzed in the Orbitrap at 50,000 resolution, with the top 8 most intense ions in the MS2 spectrum subjected to HCD fragmentation at a normalized collision energy of 55%, an AGC target of 2 × 10^5^, and a maximum injection time of 100 milliseconds (proteome) or 400 milliseconds (phospho).

### Mass spectrometry data analysis

Global and phosphorylome pSTY peptide spectra were searched using Mascot (MatrixScience, RRID: SCR_000307) against a concatenated target-decoy human database (downloaded June 2016) containing common contaminants. Searches allowed a precursor mass tolerance of 25 ppm, fragment ion tolerance of 0.5 Da, and tryptic specificity up to 2 (proteome/phospho). For global proteome analysis, the following static modifications were applied: carbamidomethyl cysteine (+57.0214), TMTPro-labeled N-terminus (+304.2071), and TMTPro-labeled lysine (+304.2071). Variable modifications included oxidized methionine (+15.9949) and TMTPro-labeled tyrosine (+304.2071). For phosphopeptide analysis, all global static and variable modifications were retained, with the addition of phosphorylation on serine, threonine, and tyrosine (+79.9663) as a variable modification. Peptide spectral matches (PSM) were filtered using linear discriminant analysis to a 2% false discovery rate (FDR) and aggregated to a 2% protein-level FDR. TMT-MS3 quantification was performed using Mojave, retaining only those PSMs with isolation specificity ≥0.5 for the final dataset. Phosphorylation data were analyzed using MSstatsTMT R package (v2.2.8; https://doi.org/10.18129/B9.bioc.MSstatsTMT; ref. 35), which performed PSM-level preprocessing to derive protein- or PTM-level quantification and perform differential abundance analysis. Log_2_ (FC) estimates and associated standard errors were calculated using a linear mixed-effects model implemented in MsStats (RRID: SCR_014353). To account for corresponding changes in protein abundance, PTM-level log_2_ (FC) values were adjusted using MSstatsPTM (v1.4.1; https://doi.org/10.18129/B9.bioc.MSstatsPTM; ref. 36), which integrates log_2_ (FC) estimates and their standard errors. Differential abundance was assessed using model-based test statistics compared with a Student *t*-distribution with protein- or PTM-specific degrees of freedom. *P* values were adjusted for multiple testing using the Benjamini–Hochberg method.

### Cell surface protein biotinylation and isolation

Cells were seeded in medium with or without 0.5 μg/mL Dox. After 4 days, plasma membrane proteins were isolated using the Pierce Cell Surface Biotinylation and Isolation Kit (Thermo Fisher Scientific, #A44390), following the manufacturer’s protocol. Briefly, cells were washed with PBS and then incubated with biotin for 10 minutes. Excess biotin was then removed, and cells were washed with TBS (25 mmol/L Tris and 0.15 mol/L NaCl, pH 7.2). Cells were scraped, collected in TBS, and centrifuged. The resulting cell pellets were lysed, and biotinylated proteins were captured on NeutrAvidin resin columns. After washing, proteins were eluted and prepared for IB analysis.

### Gene expression analysis by RT-qPCR

RNA was reverse transcribed and amplified in a QuantStudio 7 Flex thermocycler (Applied Biosystems) using the TaqMan RNA-to-CT 1-Step Kit (Thermo Fisher Scientific, #4392938). The Taqman probes are as follows: MIB1 (Thermo Fisher Scientific, #4351372, Hs01075899_m1), BMPR2 (Thermo Fisher Scientific, #4331182 Hs00176148_m1), and GAPDH (Thermo Fisher Scientific, #4331182, Hs02786624_g1).

### HIS-tag pull-down

Prior to lysis, HUVECs were treated with 10 μmol/L MG132 or 25 μmol/L CLQ for 6 hours, as indicated. Cells were lysed in Pierce IP Lysis Buffer (Thermo Fisher Scientific, #87787), and 10% to 15% of the total lysate was reserved as the input fraction. HIS-Tag Dynabeads (Thermo Fisher Scientific, #10103) were washed with 1X IP High Buffer (Active Motif, #37510) and incubated with 200 ng recombinant human BMPR2-HIS protein (Thermo Fisher Scientific, #PV6258) for 2 hours at 4°C. The BMPR2-HIS/Dynabead complexes were washed and incubated overnight at 4°C with HUVEC lysates in the presence of 10 μmol/L MG132. Pulled-down proteins were collected using a magnetic stand, washed with 1X IP High Buffer, and eluted at 70°C for 10 minutes in NuPAGE LDS Reducing Buffer (Thermo Fisher Scientific, #NP0007 and #NP0009). Samples were analyzed by IB.

### Antibodies

The following primary antibodies were used in this study. IB and immunofluorescence (IF) applications and dilutions are noted for each antibody. RRIDs are included where available; (pending) indicates identifiers not yet assigned. Anti-ACVR1 (Abcam, ab155981, IB 1:1,000), anti–β-actin (Cell Signaling Technology, #3700, IB 1:2,000, RRID: AB_2242334), anti-BMPR2 (Abcam, ab96826, IB 1:1,000; IF 1:200, RRID: AB_10679307), anti-Cas9 (Cell Signaling Technology, #14697, IF 1:500, RRID: AB_2750916), anti-CREB (Cell Signaling Technology, #9197, IB 1:1,000, RRID: AB_331277), anti-FKBP1A (Abcam, ab108420, IB 1:1,000), anti-HPRT (Thermo Fisher Scientific, PA5-22281, IB 1:1,000, RRID: AB_11153553), anti-HSP90 (BD, #610418, IB 1:1,000, RRID: AB_397798), anti-MIB1 (Abcam, ab124929, IB 1:1,000, RRID: AB_11127834; Thermo Fisher Scientific, MA5-31797, IB 1:500), anti-p21 (Cell Signaling Technology, #2947, IB 1:1,000; IF 1:200, RRID: AB_823586), anti-pSMAD1/5 (Cell Signaling Technology, #9516, IB 1:1,000, RRID: AB_491015), anti-pSMAD1/5/9 (Cell Signaling Technology, #13820, IB 1:1,000; IF 1:400, RRID: AB_2493181), anti-pSMAD2/3 (Cell Signaling Technology, #9510, IB 1:1,000; IF 1:400, RRID: AB_2193178), and anti-SMAD1 (Cell Signaling Technology, #6944, IB 1:1,000, RRID: AB_10858882; Abcam, #ab53745, IB 1:100).

Secondary antibodies used for IF were Alexa Fluor–conjugated (Invitrogen, 1:200). For IB, HRP-conjugated secondaries were from Jackson ImmunoResearch (1:5,000), and IRDye-conjugated secondaries were from Li-Cor (1:10,000).

### Image quantification

Image analysis was performed using CellProfiler Image Analysis software v4.2.7 (RRID: SCR_007358; ref. 37) to identify nuclei and quantify average nuclear fluorescence intensity per cell. Data are presented as the mean ± standard deviation (SD), with each point representing the intensity of a single nucleus.

### Statistical analyses

Data are presented as the mean ± SD. Unless otherwise noted, statistical analyses were performed using GraphPad Prism (RRID: SCR_002798). An unpaired two-tailed Student *t* test or a two-way ANOVA with Tukey *post hoc* test was used where appropriate, as indicated in the figure legends. Statistical significance is denoted in the figures with an asterisk as follows: *, *P* < 0.05; **, *P* < 0.01 and ***, *P* < 0.001; ****, *P* < 0.0001.

No formal randomization or blinding procedures were applied in this study. Experimental materials (e.g., cell lines and lysates) were assigned to groups or well positions based on alphabetical order of sample name. These studies involved *in vitro* assays without human or animal subjects. No formal power analysis was performed. Sample sizes were selected based on established norms in the field and prior experience with similar experimental designs demonstrating sufficient power to detect biologically meaningful differences.

## Results

### MIB1 scores as a strongly selective cancer dependency linked to negative regulation of TGF-β family signaling

**Figure 1.**
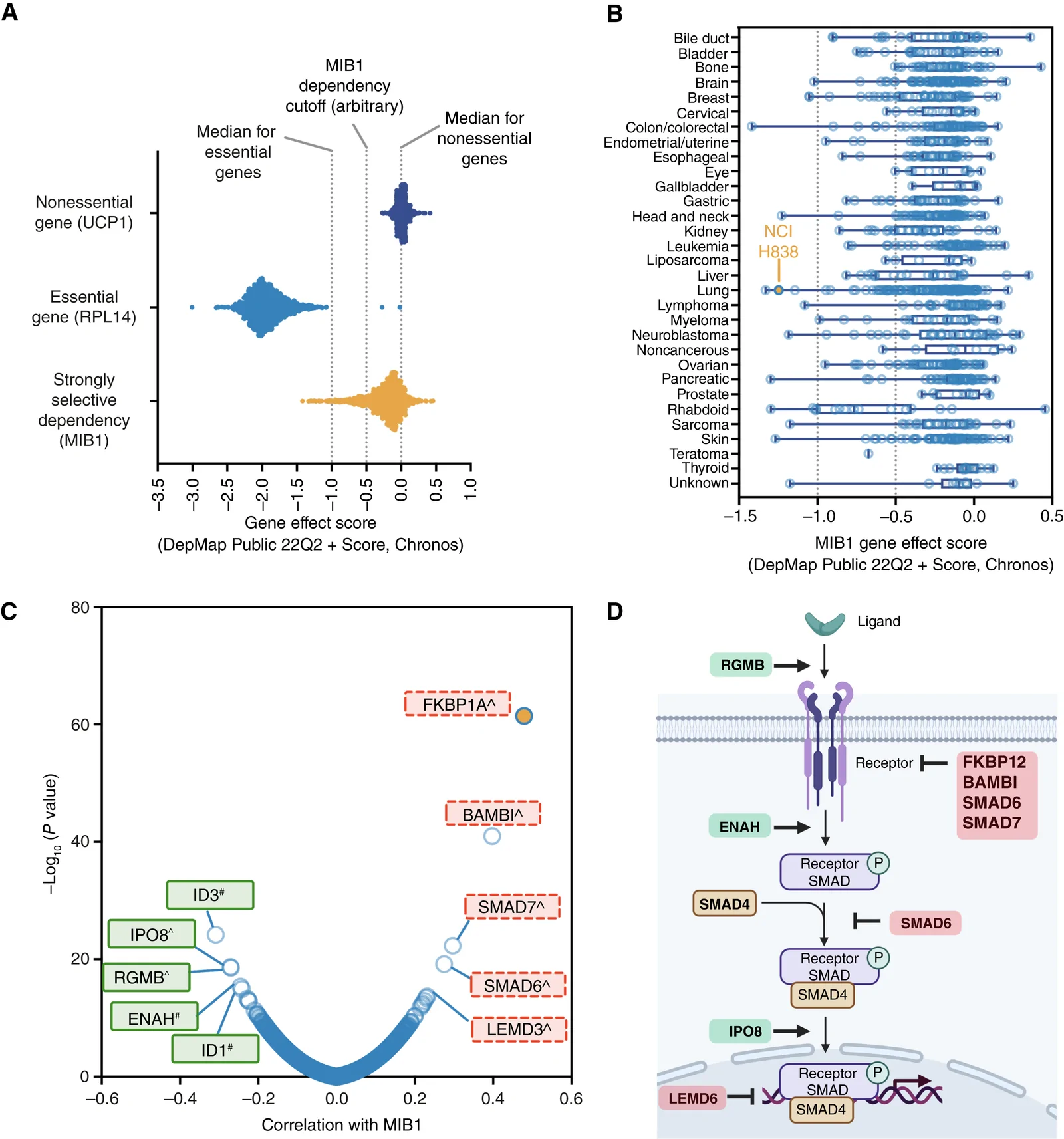
MIB1 is a strongly selective cancer dependency linked to negative regulation of TGF-β family signaling. **A,** Gene dependency scores (gene effect score) for a subset of cancer cell lines in the CRISPR (DepMap Public 22Q2, Chronos) dataset exhibiting selective MIB1 dependency. For context, dependency scores for an essential gene (*RPL14*) and a nonessential gene (*UCP1*) are also shown. **B,** MIB1 dependency scores from DepMap (Public 22Q2, Chronos) for 1,086 cancer cell lines grouped by lineage. Each circle represents an individual cell line; box-and-whisker plots summarize the distribution across lineages. **C,** Top 10 MIB1 correlates based on DepMap (Public 24Q2 Chronos and Expression). Expression correlates (#): genes whose transcript levels correlate with MIB1 dependency; codependency correlates (^): genes whose presence or absence correlates with MIB1 dependency. MIB1 anticorrelates are shown on the left (green shaded boxes, solid outlines); correlates are on the right (red shaded boxes, dashed outlines). **D,** Simplified overview of the TGF-β family signal pathway with MIB1 correlates highlighted at their points of impact. Data are presented as the mean ± SD; *n* = 3 (two-way ANOVA with Tukey correction). [**D,** Created in BioRender. Cottonham, C. (2026) https://BioRender.com/ydae6kh.]

Captured in the open-access DepMap (https://depmap.org), genome-wide CRISPR KO screening results using pooled gRNA libraries provide rich information for individual gene dependencies in more than 1,000 cancer cell lines (38). DepMap results point to MIB1 as a “strongly selective dependency” in a subset of cancer cell lines representing multiple indications (e.g., using a rigorous Chronos score cutoff of ≤ −1, MIB1 appears as a dependency in 18 cell lines; Fig. 1A and B). The most common features shared by MIB1-dependent cell lines include gene codependencies and expression levels that, surprisingly, did not highlight core components of the NOTCH pathway but rather of the TGF-β family (Fig. 1C). The top codependencies included *FKBP1A* (encoding protein FKBP12), *BAMBI*, *SMAD7*, and *SMAD6* (Fig. 1C and D), all known for their negative regulatory roles in TGF-β family signaling (1, 3). Conversely, MIB1 anticorrelates, such as *RGMB and IPO8*, transduce TGF-β family signals (Fig. 1C and D; refs. 8, 39). Numerous other associations were consistent with these examples (Fig. 1D; Supplementary Table S2) and together provocatively pointed to the hypothesis that MIB1 negatively regulates one or more signaling types within the TGF-β family.

### MIB1-dependent cells are selectively sensitive to BMP signaling

**Figure 2.**
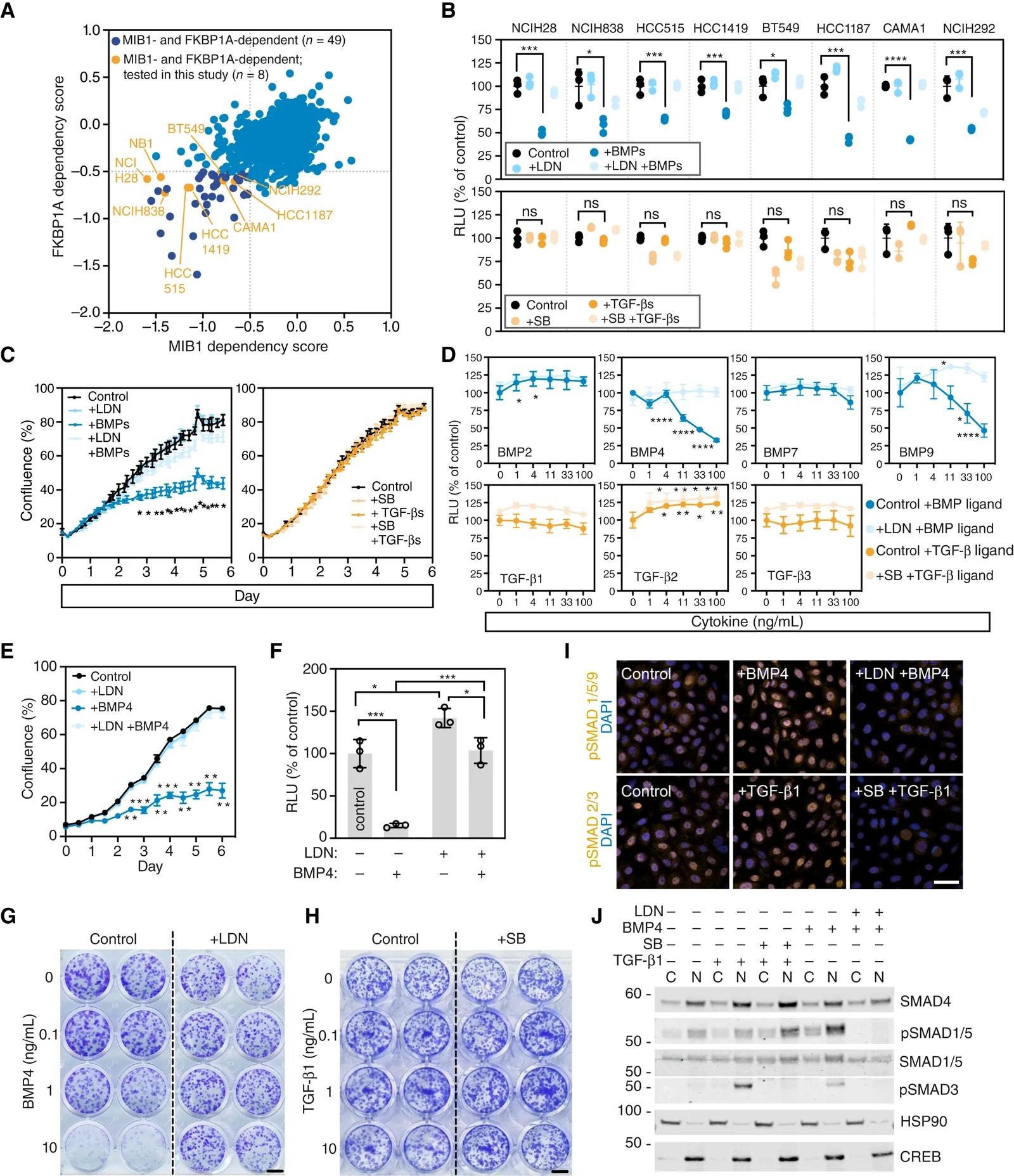
BMP ligands, but not TGF-β, inhibit growth of cell lines dependent on both MIB1 and FKBP1A. **A,** Gene dependency scores for MIB1 and FKBP1A plotted for 1,164 cancer cell lines in DepMap (Public 22Q4 Chronos). Cell lines dependent on MIB1 or FKBP1A were defined by a dependency score ≤ −0.5. **B,** MIB1- and FKBP1A-dependent cell lines were pretreated with BMP receptor inhibitor LDN (50 nmol/L) for 15 minutes followed by treatment with a cocktail of BMP ligands (top) or pretreated for 15 minutes with TGF-β receptor inhibitor SB431542 (SB, 5 μmol/L) followed by treatment with a TGF-β ligand cocktail (bottom). After 6 days, a CellTiter-Glo luminescent assay was performed. Statistical significance shown for the comparison between cytokine-treated conditions (+BMP4 or +TGF-β1) and the control group (two-way ANOVA with Tukey test, 4-group comparison). **C,** IncuCyte live-cell analysis over 6 days for NCIH838 cells treated with the BMP (left) or TGF-β (right) cocktails in the presence or absence of 50 nmol/L LDN or 5 μmol/L SB, respectively. **D,** CellTiter-Glo assay of NCIH838 cells treated with a titration of the indicated ligands over 6 days. All conditions were included in the two-way ANOVA with Dunnett’s test; statistical significance is indicated only for comparisons of +BMP4 vs. −BMP4 without LDN. **E,** IncuCyte live-cell analysis for NCIH838 cells treated with 50 nmol/L LDN, 10 ng/mL of BMP4, or both. **F,** NCIH838 cells were treated with 50 nmol/L LDN, 10 ng/mL of BMP4 or both for 7 days and then assayed by CellTiter-Glo. The results are represented as the mean ± SD; *n* = 3 (two-way ANOVA with Tukey correction). **G** and **H,** Colony formation assays for NCIH838 cells treated with titrations of BMP4 in the absence (control) or presence of LDN (**G**) or TGF-β1 in the absence (control) or presence of SB (**H**) for 7 days; scale bars, 5 mm. Representative wells shown; the experiment was repeated independently at least three times. **I,** IF staining of NCI-H838 cells for phosphorylated SMAD proteins (orange). pSMAD1/5/9 staining under control (untreated) conditions or after treatment with BMP4 (11 ng/μL), with or without 50 nmol/L LDN. pSMAD2/3 staining under control conditions or after treatment with TGF-β1 (11 ng/μL), with or without 5 μmol/L SB. Nuclei were counterstained with DAPI (blue). Scale bars, 50 μm. **J,** Immunoblot of cytoplasmic (C) and nuclear (N) fractions from NCI-H838 cells pretreated with 1 μmol/L LDN or 5 μmol/L SB for 24 hours and then stimulated with 10 ng/mL BMP4 or TGF-β1 for 1.5 hours to detect pSMAD proteins. CREB and HSP90 were used as loading controls for nuclear and cytoplasmic fractions, respectively. For all panels, *, *P* < 0.05; **, *P* < 0.01 and ***, *P* < 0.001; ****, *P* < 0.0001; “ns,” not significant.

To study this link between MIB1 and TGF-β family signaling, we identified 49 cell lines that depended (Chronos score ≤ −0.5) on both MIB1 and FKBP1A (Fig. 2A; Supplementary Table S3), the top MIB1 codependency (Fig. 1C) and a well-characterized gatekeeper of TGF-β family signaling that blocks receptor phosphorylation (40, 41). Such criteria should enrich for lines in which MIB1 dependency reflects TGF-β regulation as opposed to other mechanisms that could manifest in MIB1 dependency. To ascertain the TGF-β subfamily with which MIB1 dependency correlated, we selected a subpanel of these 49 lines, comprising four breast and four lung lines that express various BMP and TGF-β receptors (Supplementary Fig. S1A). We treated the subpanel with cocktails of BMP or TGF-β ligands. BMP but not TGF-β ligands inhibited the growth of each of the lines, and cotreatment with an inhibitor of BMP type I receptors (“LDN”) rescued this growth defect (Fig. 2B). In NCIH838 lung cancer cells, BMP ligands induced growth inhibition as early as 2 days after treatment (Fig. 2C). We confirmed that LDN rescued this growth inhibition and that growth was insensitive to TGF-β ligands (Fig. 2C). To pinpoint the functionally relevant BMP ligand(s), we titrated each cytokine and found that BMPs 4 and 9, but not BMPs 2 or 7, inhibited growth in a manner that was again rescued by LDN and insensitive to individual TGF-β ligands (Fig. 2D–F). Using a colony formation assay as a distinct test of growth, we found that BMP4 reduced colony number and size in a manner that remained dose-dependent, rescued by LDN, and insensitive to TGF-β1 (Fig. 2G and H). Both BMP4 or TGF-β1 stimulated pSMAD nuclear accumulation (Fig. 2I and J; Supplementary Fig. S1B), thus controlling for ligand activity and demonstrating that the cells are competent to transduce both BMP and TGF-β signals. Together, our data indicate that the MIB1- and FKBP1A-dependent cell lines are functionally sensitive to specific BMPs 4 and 9 but not TGF-β isoforms.

### MIB1 loss phenocopies BMP tumor-suppressor function

**Figure 3.**
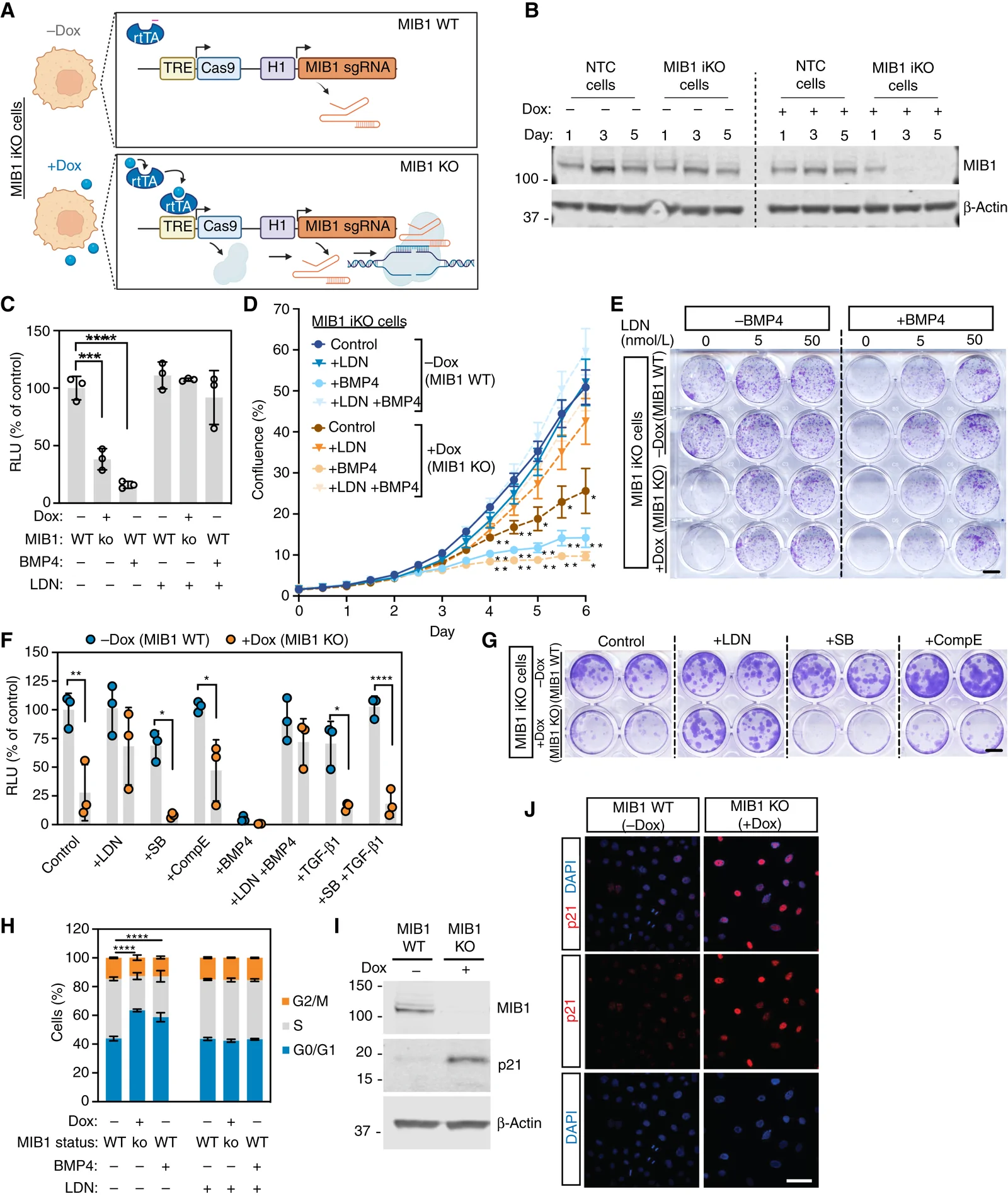
MIB1 KO mirrors BMP-mediated suppression of cell proliferation and cell-cycle progression. **A,** Overview of the all-in-one CRISPR/Cas9 expression construct to generate MIB1-iKO. MIB1 is targeted by Cas9 following 0.5 μg/mL Dox addition. **B,** MIB1 protein was evaluated by IB after a time course of 0.5 μg/mL Dox treatment in clone 2.1. **C–E,** MIB1-iKO cells were treated with 0.5 μg/mL (Dox), 10 ng/mL BMP4, or both, with or without 50 nmol/L LDN. **E,** includes 5 nmol/L LDN. **C,** Cell viability was assessed using CellTiter-Glo on day 12 (*n* = 3). Statistical analysis was performed using two-way ANOVA with Tukey *post hoc* test. **D,** Cell growth was monitored by IncuCyte live-cell imaging over 6 days (*n* = 3). Two-way ANOVA with Tukey test was used for statistical analysis. **E,** Colony formation assay performed on day 7. Representative wells shown from three independent experiments. Scale bars, 5 mm. **F** and **G,** MIB1-iKO cells were treated with the BMP inhibitor LDN (50 nmol/L), TGF-β inhibitor SB (5 μmol/L), or Notch inhibitor Compound E (CompE, 1 μmol/L) and stimulated with 10 ng/mL BMP4 or 10 ng/mL TGF-β1 with or without Dox (0.5 μg/mL). **F,** A CellTiter-Glo assay was performed on day 14. The results are represented as the mean ± SD; *n* = 3 (two-way ANOVA with Tukey correction). **G,** Colony formation assay performed on day 14. Representative images shown from three independent experiments. Scale bars, 5 mm. **H,** MIB1-iKO cells were treated with combinations of Dox (0.5 μg/mL), BMP4 (10 ng/mL), and LDN (50 nmol/L), as indicated. After 4 days, EdU was incorporated for 1 hour and detected by flow cytometry to assess cell-cycle distribution. Data are from three independent experiments. Statistical significance was determined for all cell-cycle phases using two-way ANOVA with Tukey *post hoc* test; *P* values are shown for G0/G1. **I,** Immunoblot of MIB1 and p21 protein levels after 3 days of 0.5 μg/mL Dox treatment. **J,** IF to detect the subcellular localization of p21 (red). DAPI staining was used to visualize nuclei (blue). Scale bars, 50 μm. Representative images from three independent experiments are shown. For all panels, *, *P* < 0.05; **, *P* < 0.01 and ***, *P* < 0.001; ****, *P* < 0.0001. [**A,** Created in BioRender. Cottonham, C. (2026) https://BioRender.com/9nig4ft.]

To further examine the impact of MIB1 depletion, we developed an inducible MIB1 gene KO system by engineering NCIH838 cells with Dox-inducible CRISPR/Cas9 editing constructs (Fig. 3A). IB analyses after 3 days of Dox induction in clonal cell lines (MIB1-iKO cells) revealed a substantial reduction in MIB1 protein levels, dependent on Dox and MIB1 gRNAs (Fig. 3B; Supplementary Fig. S2A). Using a variety of growth assays, we found that MIB1 depletion mimicked BMP4 addition, with both perturbations inducing growth inhibition that was rescued by LDN (Fig. 3C–E) and phenocopied by FKBP1A KO (Supplementary Fig. S2B and S2C). Although these cells express receptors and ligands from both the Notch (Supplementary Fig. S2D) and core TGF-β pathways (Supplementary Fig. S1A), we found that treatment with either a γ-secretase inhibitor (CompE) to block Notch or SB to inhibit TGF-β receptors did not affect growth or colony formation, regardless of MIB1 status (Fig. 3F and G). Flow cytometry cell-cycle analyses revealed that BMP4 treatment or MIB1 KO similarly (i) arrested cells in the G0/G1 phases, (ii) decreased the fraction of cells in S-phase (Fig. 3H), and (iii) increased the level and nuclear localization of the cell-cycle protein p21^Cip1/Waf1^ (Fig. 3I and J; Supplementary Fig. S2E), consistent with previous reports of BMP-induced cell-cycle arrest through p21^Cip1/Waf1^ (42). Together, these findings show that MIB1 loss phenocopies BMP tumor-suppressive function and highlight the importance of suppressing BMP signals, but not signals from Notch or TGF-β, to maintain NCIH838 growth and cell-cycle progression.

### MIB1 represses BMP signaling

**Figure 4.**
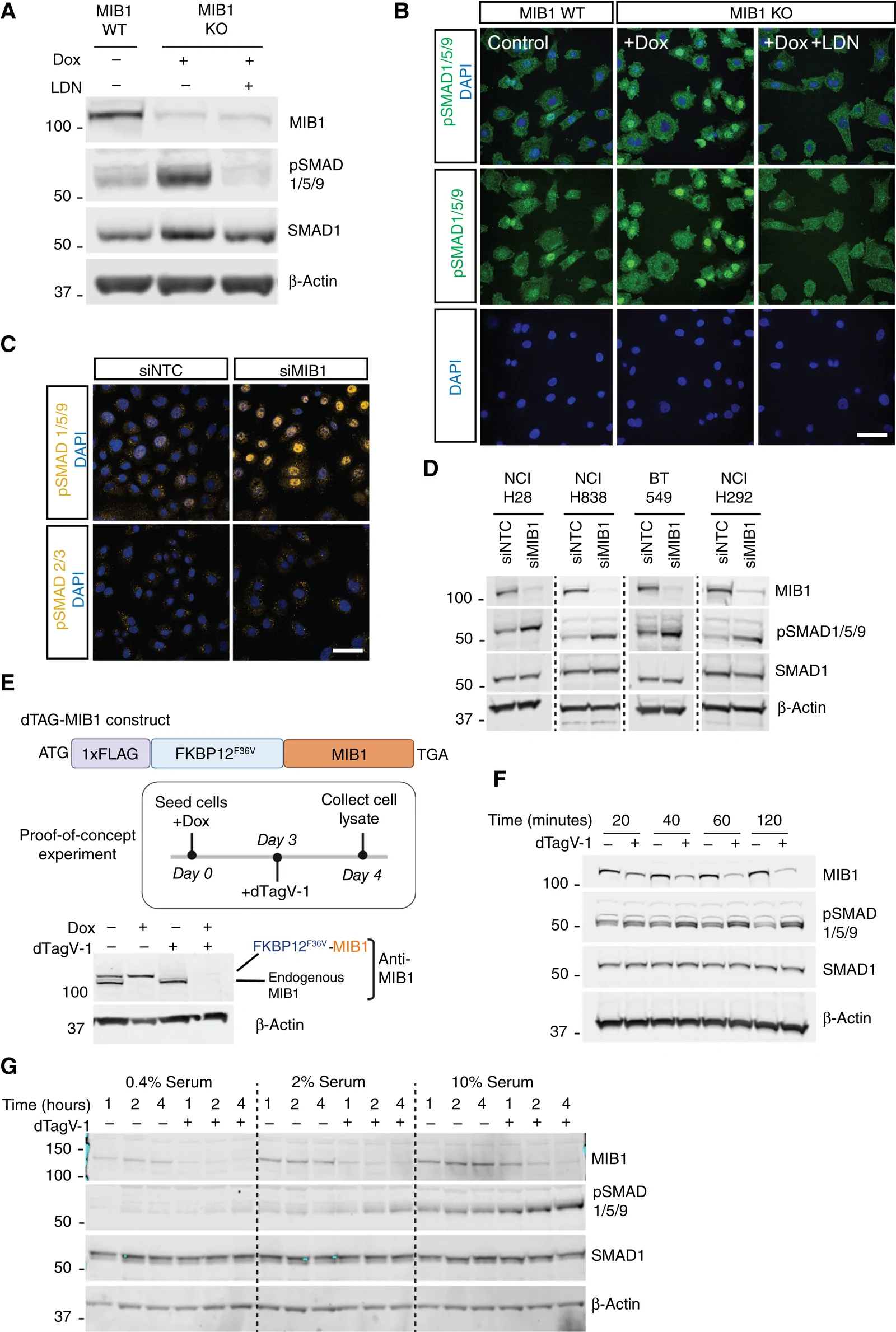
Loss of MIB1 enhances BMP signaling activity. **A,** Immunoblot analysis of pSMAD1/5/9 levels in MIB1-iKO cells treated with Dox (0.5 μg/mL) and BMP inhibitor LDN (50 nmol/L) for 3 days. β-Actin served as the loading control. **B,** IF analysis of pSMAD1/5/9 (green) with DAPI (blue) nuclear staining in cells treated as described in **A**; scale bars, 50 μm. **C,** Phospho-SMAD1/5/9 (orange) or pSMAD2/3 (orange) were evaluated by IF in NCI-H838 cells transfected with MIB1 or nontargeting control (NTC) siRNA for 48 hours, with DAPI nuclear staining (blue); scale bar, 50 μm. **D,** Immunoblot analysis of pSMAD1/5/9 in additional MIB1- and FKBP1A-dependent cancer cell lines transfected with MIB1 or NTC siRNA for 48 to 72 hours. β-Actin served as the loading control. **E,** Top, schematic of the dTAG-MIB1 construct that expresses a FKBP12^F36V^–MIB1 fusion protein (dTAG-MIB1, Cas9-resistant) that is degraded upon addition of dTagV-1 ligand. **E,** Bottom, proof-of-concept experiment: NCI-H838 MIB1-iKO cells engineered to express dTAG-MIB1 and were treated with Dox (0.5 μg/mL), dTagV-1 (100 nmol/L), or a combination of both. After 24 hours, MIB1 protein levels were analyzed by IB. β-Actin served as the loading control. **F,** Time-course analysis using IB to assess MIB1 and pSMAD1/5/9 protein levels after different durations of dTagV-1 (100 nmol/L) in dTAG-MIB1 cells. β-Actin served as the loading control. **G,** pSMAD1/5/9 protein level in dTAG-MIB1 cells grown in 0.4%, 2%, or 10% serum 18 hours prior to dTagV-1 ligand treatment. β-Actin served as the loading control. Data are representative of three independent experiments. [**E** (top), Created in BioRender. Cottonham, C. (2026) https://BioRender.com/jbpsnmi.]

We assessed the consequences of MIB1 loss on downstream signaling events in NCIH838 cells using antibodies to detect phosphorylated SMADs 1, 5, and 9 (pSMAD1/5/9) or phosphorylated SMADs 2 and 3 (pSMAD2/3), well-documented markers of active BMP or TGF-β signaling, respectively (43, 44). IB and IF staining revealed low but reproducibly detectable pSMAD1/5/9 protein and nuclear localization in WT cells under standard growth conditions, thus benchmarking a weak level of baseline BMP signaling, perhaps induced by BMP ligands present in the culture medium (Fig. 4A and B). MIB1 depletion robustly increased pSMAD1/5/9 levels and nuclear localization in an LDN-inhibitable manner with little effect on overall levels of SMAD1 (Fig. 4A and B; Supplementary Fig. S3A), consistent with MIB1 loss increasing BMP signal strength. These effects were selective for BMP and not TGF-β signaling because siRNA knockdown of MIB1 increased levels of pSMAD1/5/9 but not pSMAD2/3 (Fig. 4C; Supplementary Fig. S3B). MIB1 knockdown in three additional MIB1- and FKBP1A-dependent lung and breast cancer cell lines similarly increased SMAD1/5/9 phosphorylation (Fig. 4D), suggesting that MIB1 suppression of BMP signaling is a generalizable phenomenon in multiple cancer lineages.

To examine MIB1 loss on immediate-early BMP signaling, we generated an NCI-H838 MIB1-dTAG cell line (45) for rapid, inducible, proteasome-dependent MIB1 degradation, bypassing the extended timeline of CRISPR/Cas9 deletions (Fig. 4E). Treatment of MIB1-dTAG cells with the dTag ligand, dTagV-1, induced marked MIB1 degradation within 60 to 120 minutes, compared with the 72 hours required for CRISPR-mediated MIB1 loss in the NCIH838 MIB1-iKO cells (Figs. 3A and 4F). MIB1 degradation reduced colony formation (Supplementary Fig. S3C) and increased pSMAD1/5/9 levels (Fig. 4F). Additionally, SMAD1/5/9 phosphorylation closely tracked with serum concentration, in which higher serum levels correlated with increased phosphorylation (Fig. 4G; Supplementary Fig. S3D), indicating that the activating signal for BMP signaling is inherently present in the serum component, and MIB1 loss serves to derepress and potentiate this constitutive BMP pathway activity.

**Figure 5.**
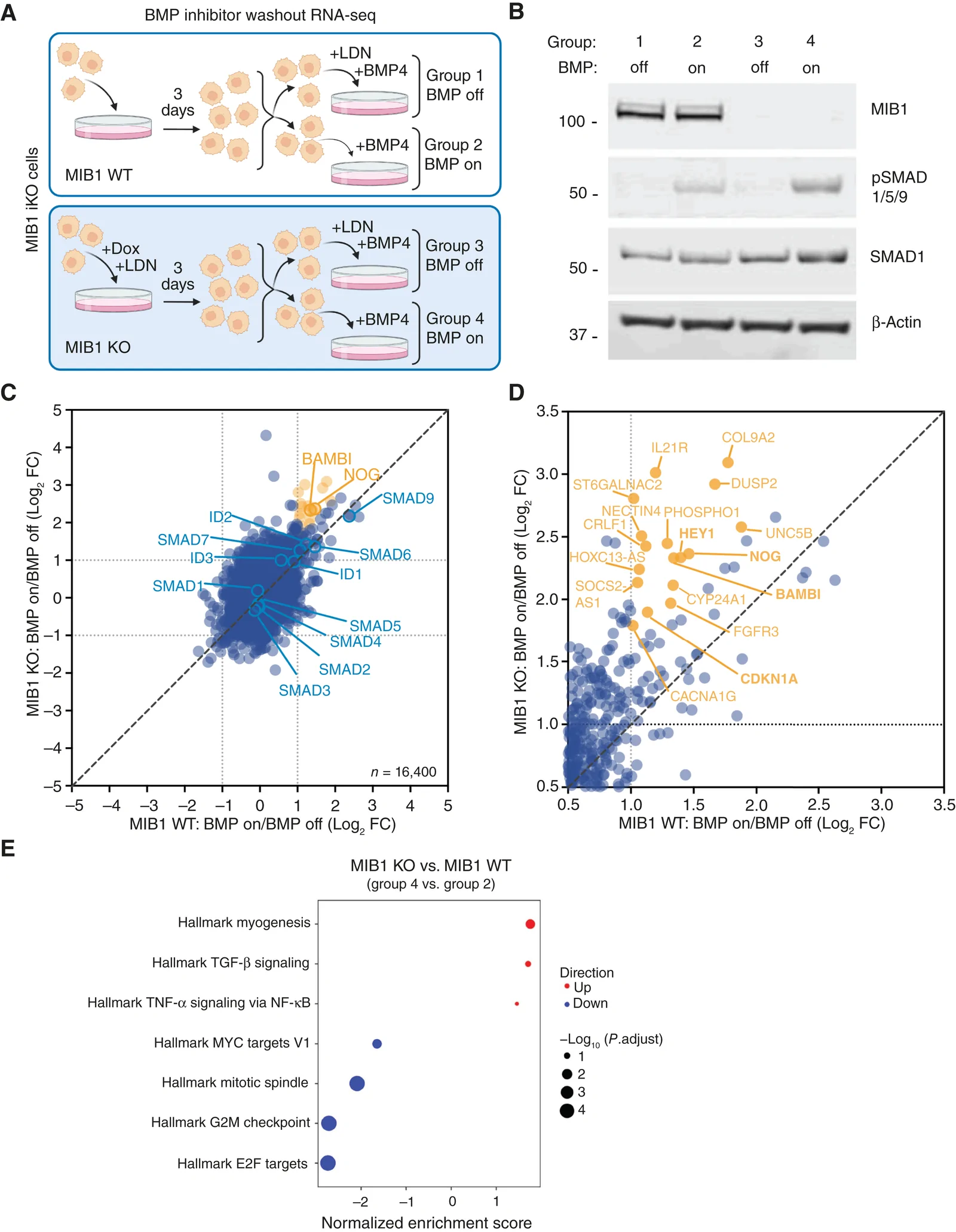
Loss of MIB1 amplifies BMP target gene expression. **A,** Overview of the BMP inhibitor washout experiment for RNA-seq. MIB1 KO was induced by Dox (0.5 μg/mL) under BMP-off conditions (+LDN, 50 nmol/L). After 3 days, cells were split equally into medium containing BMP4 (10 ng/mL) with or without continued LDN (50 nmol/L) treatment, generating groups 1 to 4 as indicated. RNA was harvested 24 hours later for sequencing analysis. **B,** In parallel with RNA collection, BMP pathway activity was assessed by IB detection of SMAD1/5/9 phosphorylation 24 hours after washout of the BMP inhibitor. This confirmed effective pathway suppression and reactivation. **C,** RNA-seq analysis of gene expression changes following BMP inhibitor washout (*n* = 3 per group; 16,400 genes analyzed). The dashed line indicates genes with equivalent FC upon BMP reactivation (BMP-on/BMP-off) in both MIB1 KO and MIB1 WT cells. Potentiated BMP target genes are labeled in bold orange; other BMP target genes and pathway components are indicated with blue labels. **D,** Zoomed-in view of the region in **C** highlighting potentiated genes (orange), with BMP target genes in bold orange. **E,** GSEA of RNA-seq data from the BMP inhibitor washout experiment (**C** and **D**), comparing group 4 (MIB1 KO, BMP-on) with group 2 (MIB1 WT, BMP-on). Gene sets were derived from the Hallmark database. [**A,** Created in BioRender. Cottonham, C. (2026) https://BioRender.com/5qnb2jv.]

Next, we determined how MIB1 depletion affected BMP signaling downstream of pSMAD1/5/9. To do this, we analyzed transcriptional changes using RNA-seq, using a LDN wash-out approach to maintain BMP signal repression during the 3 days required for Cas9 editing, before triggering a precisely timed signal release through LDN washout. Specifically, we grew MIB1-iKO or control cells for 72 hours in the presence of LDN while treating with Dox to knock out MIB1; we then refreshed with medium that either contained LDN to maintain “pathway off” conditions or lacked LDN to enable “pathway on” conditions (Fig. 5A). IB 1 hour after washout showed basal and enhanced SMAD1/5/9 phosphorylation in WT and MIB1-iKO cells, respectively, thus validating the approach (Fig. 5B). Four treatment groups were analyzed: (i) MIB1 WT with BMP-off, (ii) MIB1 WT with BMP-on, (iii) MIB1 KO with BMP-off, and (iv) MIB1 KO with BMP-on. A four-way comparison of MIB1 KO (groups 3 and 4) versus control (groups 1 and 2) revealed a notable overlap in the transcriptional profiles of MIB1 WT and KO cells when comparing BMP-on and BMP-off states (Fig. 5C and D). MIB1 KO potentiated the BMP response for a subset of genes, including *BAMBI* and *Noggin* (*NOG*), recognized negative regulators of the BMP pathway (3), and *CDKN1A*, a cell-cycle regulator (Fig. 5D; Supplementary Fig. S4A; ref. 46). When each BMP condition was examined individually, *BAMBI* and *NOG* were increased in MIB1 KO cells under BMP-on conditions, but not under BMP-off conditions, consistent with their roles as BMP transcriptional targets (Supplementary Fig. S4A). Interestingly, *SMAD9*, a transcriptional target of BMP that also functions in negative feedback (47), was increased in the BMP-off condition despite pharmacologic inhibition of BMP receptor signaling (Supplementary Fig. S4A). These effects on gene expression were further supported by an independent RNA-seq experiment under basal BMP signaling, in which MIB1 KO similarly increased *SMAD9* expression and modestly affected other BMP targets, including *ID* proteins and *NOG*, although these changes did not reach statistical significance (Supplementary Fig. S4B). GSEA showed increased expression of BMP pathway genes (Hallmark TGF-β signaling) and simultaneous decreased expression of cell cycle–related pathways when control and KO cells were compared under the BMP-on condition (group 4 vs. group 2; Fig. 5E). These findings collectively suggest a consistent pattern of gene expression changes in response to BMP4 treatment, whether in control or MIB1 KO cells.

### A MIB1 ligase domain is required for negative regulation of BMP signaling

**Figure 6.**
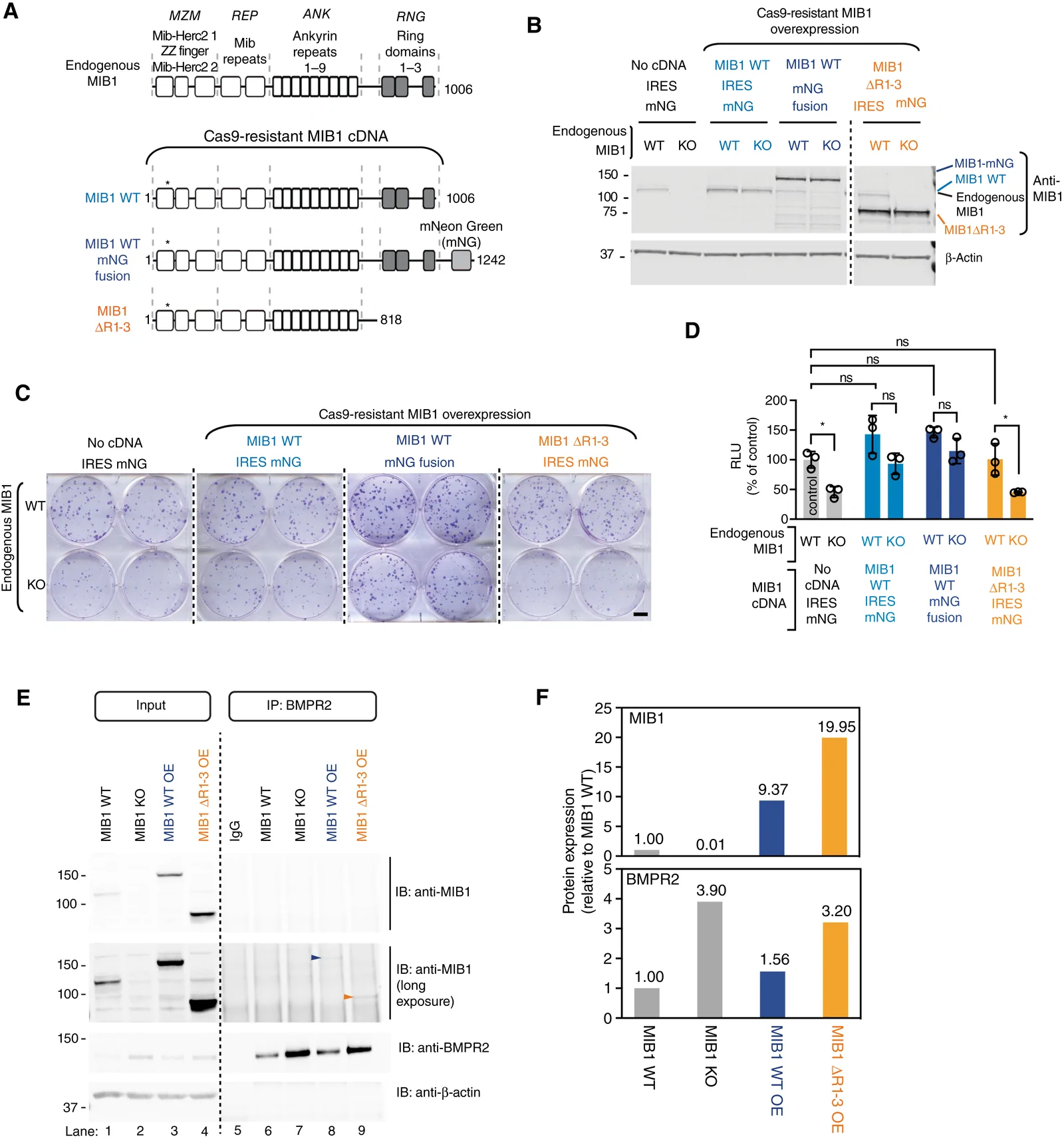
The ligase domains of MIB1 are essential for its regulatory function in BMP. **A,** Top, schematic (modified from (20)) of the endogenous MIB1 coding region, with exons shown as boxes and protein domains indicated. **A,** Bottom, Cas9-resistant MIB1 cDNA constructs, containing mutated PAM sequences (asterisks), express either WT MIB1 cDNA (MIB1 WT, blue) or MIB1 cDNA with all three RING domains deleted (MIB1ΔR1-3, orange). **B,** Immunoblot analysis of MIB1 protein levels in MIB1-iKO cells that were engineered to stably express Cas9-resistant MIB1 WT or MIB1 ΔR1-3 after 3 days of Dox (0.5 μg/mL) treatment. β-Actin served as the loading control. **C–E,** MIB1-iKO cells overexpressing MIB1 WT or MIB1ΔR1–3 were treated with Dox (0.5 μg/mL) for the indicated time points. **C,** Colony formation assay after 14 days of Dox treatment; scale bars, 10 mm. Images are representative of three independent experiments. **D,** CellTiter-Glo assay after 7 days of Dox treatment. Data represent three independent experiments. Two-way ANOVA with Tukey test; *, *P* < 0.05. “ns” denotes nonsignificant differences between no MIB1 cDNA cells and those expressing MIB1 constructs. **E,** Immunoblot analysis of BMPR2 immunoprecipitates from MIB1-iKO cells. Lane labels indicate MIB1 WT (no cDNA, −Dox), MIB1 KO (no cDNA, +Dox; Dox-induced KO of endogenous MIB1), MIB1 WT OE (MIB1-mNeonGreen expressed in MIB1 KO cells), and MIB1ΔR1-3 OE (MIB1ΔR1-3-IRES-mNeonGreen expressed in MIB1 KO cells). Cell lysates were subjected to IP with anti-BMPR2 antibody or IgG control, followed by IB with the indicated antibodies. Input lysates are shown for comparison. A longer exposure of the MIB1 immunoblot is included to facilitate detection of MIB1 in BMPR2 immunoprecipitates. Blue and orange arrowheads indicate BMPR2-associated MIB1 WT OE and MIB1ΔR1-3 OE, respectively. β-Actin is shown as an input and specificity control. **F,** Densitometric quantification of MIB1 and BMPR2 protein levels from the input immunoblots shown in **E**. Signals were normalized to β-Actin and expressed relative to the MIB1 WT (no cDNA, −Dox) control.

MIB1 contains three E3 ligase RING domains, the third of which is required for MIB1 function in Notch signaling (15, 18, 22). To determine whether these domains are required for BMP negative regulation, we performed genetic complementation rescue experiments by engineering NCIH838 MIB1-iKO cells to express Cas9-resistant cDNAs that express either WT MIB1 (MIB1-WT), WT MIB1 fused to the mNeonGreen protein (MIB1 WT mNG fusion), or mutant MIB1 lacking all three RING domains (MIB1-ΔR1-3; Fig. 6A). IB revealed that endogenous MIB1 expression was effectively reduced following KO in these engineered lines and that the rescue constructs overexpressed MIB1 protein (Fig. 6B). Both WT expression constructs partially rescued the MIB1 KO–induced defects in colony formation and viability assays, whereas MIB1-ΔR1-3 expression did not (Fig. 6C and D). In both the presence or absence of endogenous MIB1, WT but not ΔR1-3 overexpression elevated growth (Fig. 6D). Growth values (RLU) were lower in all groups in the KO background relative to the WT background, likely reflecting overall expression levels of MIB1 (Fig. 6D). Using immunoprecipitation (IP) to enrich for BMPR2 [Fig. 6E (lanes 5 and 6)], we confirmed that MIB1 KO increased BMPR2 protein levels [Fig. 6E (lanes 6 and 7)] and, more importantly, observed that reexpression of WT but not ΔR1-3 reduced BMPR2 levels to toward those seen in cells expressing only endogenous MIB1 (Fig. 6E and F). Thus, the MIB1 RING domains—and by extension MIB1 E3 ligase activity—appear required for MIB1 to exert its effects on growth and BMPR2 levels.

### MIB1 negatively regulates BMPR2 protein levels in cancer and endothelial cells

**Figure 7.**
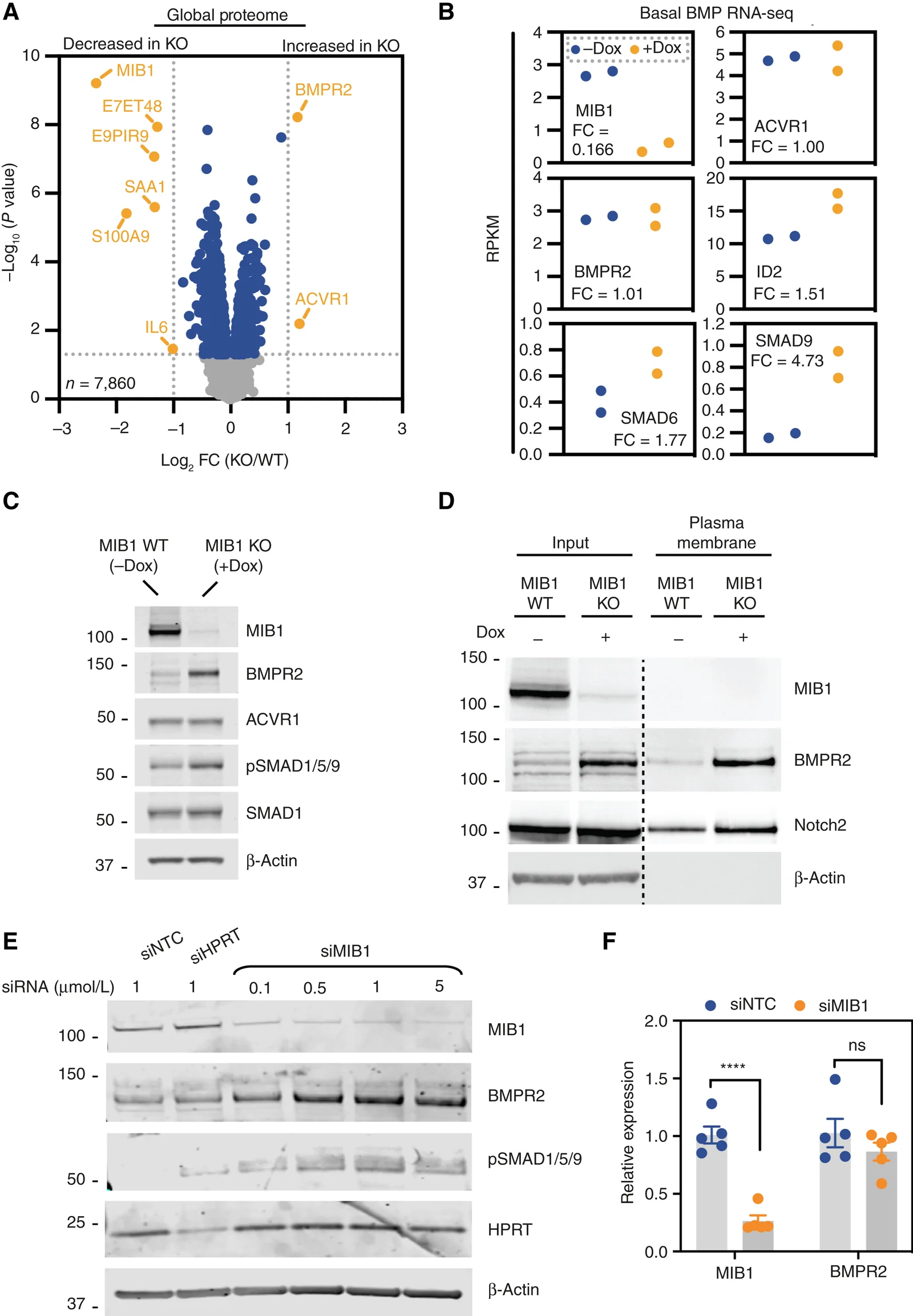
MIB1 negatively regulates BMPR2 protein levels. **A,** Global proteomic analysis by mass spectrometry of MIB1-iKO cells treated with Dox (0.5 μg/mL) for 3 days (*n* = 3). Volcano plot displays proteins with >2-fold change and statistical significance based on −log_10_ (*P* value) for MIB1 KO (+Dox) vs. MIB1 WT (−Dox). A total of 7,860 proteins were quantified. Orange points indicate proteins with >2-fold change and meeting the significance threshold; blue points indicate statistically significant proteins with <2-fold change; gray points did not meet the significance criteria. **B,** RNA-seq expression (RPKM) of selected genes from MIB1-iKO cells treated with Dox (0.5 μg/mL) for 3 days under basal BMP signaling conditions. *BMPR2* and *ACVR1* mRNA levels do not show notable changes. Each plot shows two biological replicates per condition (*n* = 2). FC was calculated from RPKM values comparing +Dox, KO vs. −Dox, WT. No statistical analysis was performed. RPKM, normalized reads per kilobase gene model per million total reads. **C,** Immunoblot analysis of BMP pathway proteins in MIB1-iKO cells treated with Dox (0.5 μg/mL) for 3 days. β-Actin served as the loading control. **D,** Immunoblot analysis of biotinylated cell surface proteins from MIB1-iKO cells treated with or without Dox (0.5 μg/mL) for 4 days, showing BMPR2 at the plasma membrane in the absence of MIB1. Immunoblot (**E**) and qPCR (**F**) analyses for HUVECs transfected with siRNA targeting MIB1, HPRT (positive control), or a nontargeting control (NTC) for 72 hours. RNA expression normalized to GAPDH. Experiments were repeated with at least three donors. *P* values were calculated using an unpaired Student *t* test; ****, *P* < 0.0001; and “ns,” not significant.

The E3 ligase function of MIB1 has been described as catalyzing monoubiquitination events that control substrate function rather than turnover (16–18, 48). However, given that ubiquitination commonly marks proteins for degradation and that our IB experiments indicated that MIB1 loss correlated with higher expression of BMPR2 protein (Fig. 6E), we analyzed MIB1-iKO cells by mass spectrometry to assess how MIB1 affects protein levels, as well as protein phosphorylation across the proteome (Supplementary Fig. S5A and S5B). The experiment was a technical success as Dox treatment reduced MIB1 protein levels (Fig. 7A; Supplementary Table S4) and increased SMAD1 phosphorylation (Supplementary Fig. S5B), consistent with loss of MIB1 expression and a gain of BMP signaling activity. Notably, MIB1 loss elevated the overall level of only two proteins by greater than twofold: BMPR2 and ACVR1, confirming the IB result noted above and demonstrating that MIB1 loss selectively increased only a BMP type II and type I receptor (Fig. 7A; ref. 3). Expression of transcripts encoding these receptors was unchanged (Fig. 7B), highlighting a posttranscriptional control mechanism. Additional IB confirmed that MIB1 loss increased BMPR2 levels and that this increase correlated with an increase in BMP signaling, as assessed by increased levels of pSMAD1/5/9 (Fig. 7C), reinforcing the reliability of the mass spectrometry analysis. In immunoblots of cellular fractions enriched for plasma membrane components, this increase of BMPR2 protein expression was particularly clear, in contrast to little or no change in the control Notch2 protein (Fig. 7D).

Given that BMP signaling affects diseases in addition to cancer, we sought to determine whether MIB1 negative regulation of BMP signaling was important in other contexts. Given the key role that reduced BMPR2 expression and activity play in pulmonary arterial hypertension (PAH), a critical therapeutic indication in need of new treatments, we asked whether MIB1 also suppressed BMPR2 levels in endothelial cells. We transfected human primary endothelial cells with siRNAs targeting MIB1 and found that MIB1 protein levels inversely correlated with levels of BMPR2 protein but not mRNA (Fig. 7E and F), consistent with our findings of posttranscriptional control in cancer cells. Together, our results reveal that MIB1 negatively regulates BMP signals by controlling BMP receptor protein levels in both cancer and endothelial cells.

To gain additional insights into molecular mechanisms through which MIB1 might regulate BMP signaling and control BMPR2 levels, we analyzed changes in the global phosphoproteome (Supplementary Fig. S5A). We were particularly interested in identifying changes downstream of MIB1 but upstream of BMP signaling; in other words, we focused on changes that were observed whether BMP signaling was at the basal, inhibited, or ligand-driven level (Supplementary Fig. S5A). As a control, we found that SMAD1 phosphorylation at S463/S465 increased upon MIB1 loss under basal conditions as well as after BMP stimulation, consistent with our IB results and validating the technical success of the phosphoproteomics analysis (Supplementary Fig. S5B). The only protein that showed phosphorylation changes that depended on MIB1 but not BMP signaling was RASA2, a RAS-GTPase–activating protein (RAS-GAP; refs. 49, 50). MIB1 loss increased RASA2 phosphorylation at S598 and T600 to similar extents under basal, inhibited, and ligand-driven conditions (Supplementary Fig. S5B). Intriguingly, RASA2 has been linked to Noonan syndrome (51), a RAS-opathy that carries increased risk for certain cancers, and to PAH, a BMPR2-related disease (52). Our results show that MIB1 regulates phospho-Rasa2 independent of BMP signaling and could potentially act between MIB1 and BMPR2 to control BMP signaling output. This control mechanism may be important in both cancer and other diseases.

### MIB1 and BMPR2 associate in cancer and endothelial cells

**Figure 8.**
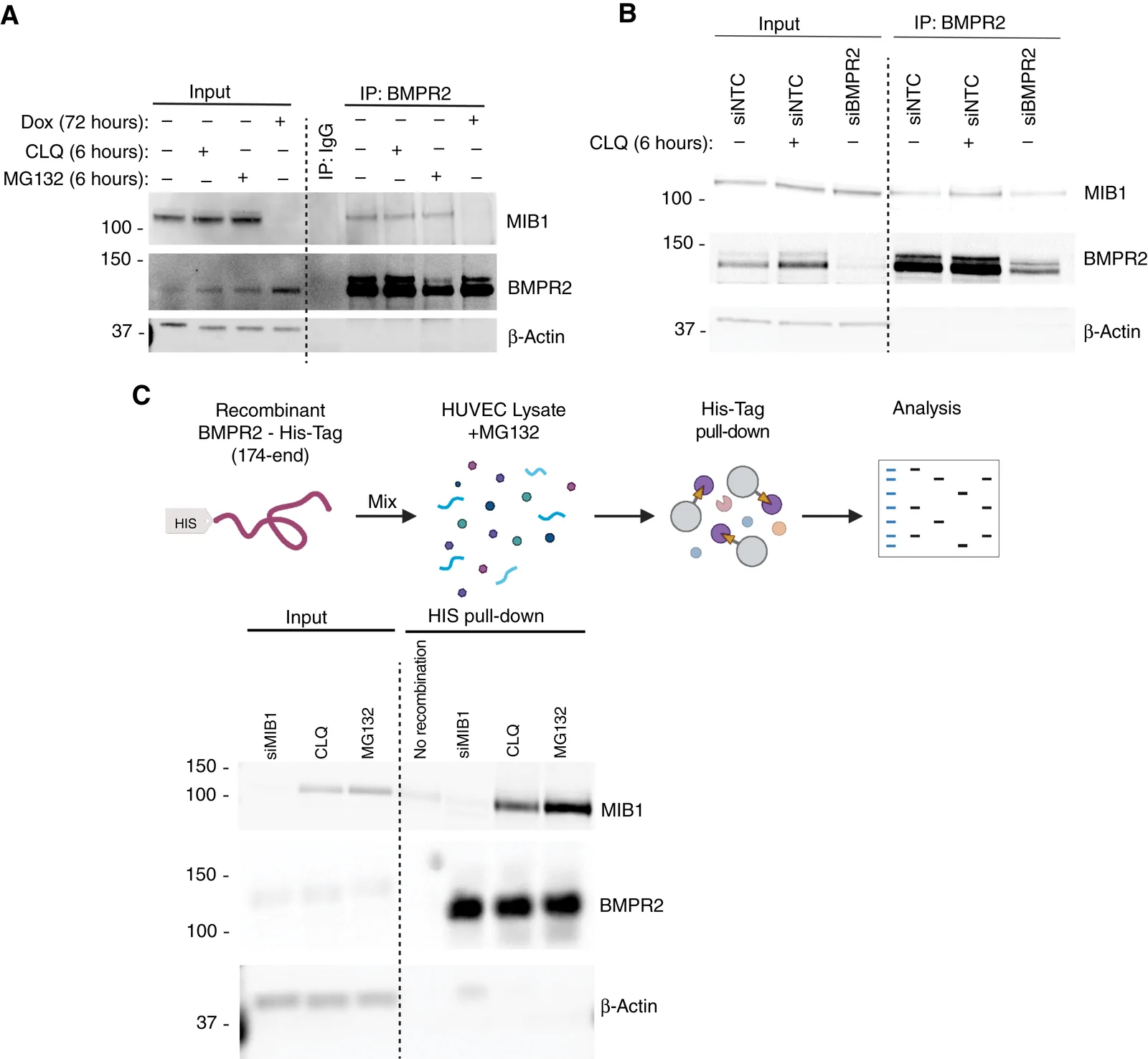
MIB1 and BMPR2 associate in cancer and endothelial cells. **A,** Co-IP of endogenous BMPR2 and MIB1 from NCIH838 cells treated with CLQ (25 μmol/L) or MG132 (10 μmol/L) for 6 hours prior to lysis to stabilize BMPR2 protein levels. IB confirmed co-precipitation of MIB1. Dox, used to induce KO of MIB1. **B,** Co-IP of endogenous BMPR2 and MIB1 from HUVECs transfected with control or BMPR2 siRNA for 72 hours. IP and IB were performed as described in **A**. **C,** Schematic (top) illustrates the experimental workflow for pull-down of endogenous MIB1 from HUVEC lysates using recombinant His-tagged BMPR2. Association of MIB1 with BMPR2 was verified by IB.

To explore whether MIB1 regulates BMPR2 protein levels through a direct or indirect mechanism, and because MIB1 and BMPR2 have been identified as putative interaction partners (53), we asked whether MIB1 and BMPR2 physically associate. Specifically, anti-BMPR2, but not isotype control antibody, immunoprecipitated MIB1 in H838 (Fig. 8A) and HUVEC (Fig. 8B) cells. RNA silencing of MIB1 or BMPR2 expression significantly reduced co-IP, supporting the conclusion that the two proteins physically associate (Fig. 8A and B). Consistent with a short BMPR2 protein half-life—estimated at less than 2 hours (54)—we found that stabilizing BMPR2 using CLQ or MG132 to inhibit lysosomal or proteasomal degradation, respectively, enhanced MIB1-BMPR2 co-IP in some contexts but was not necessary to detect the complex (Fig. 8). BMPR2 pull-down assays similarly showed a specific interaction between the two proteins, in this case recombinant His-tagged BMPR2 bound to endogenous MIB1 in endothelial cell lysates (Fig. 8C). Together, these data demonstrate a reproducible physical interaction between MIB1 and BMPR2 in both cancer and endothelial cells, highlighting its relevance in diverse cell types, including one relevant to normal physiologic functions.

## Discussion

Large-scale genomic studies aimed at identifying cancer dependency genes—genes crucial for cancer cell proliferation and survival—have become an important means to discover targets for cancer therapeutic development. The DepMap, resulting from genome-wide loss-of-function screens involving more than 1,000 cancer cell lines (38), reveals MIB1 as a “strongly selective” cancer dependency gene, indicating that a subset of cell lines is particularly sensitive to MIB1 loss. Intriguingly, the top-correlated codependencies with MIB1 did not highlight Notch signaling components, even though MIB1 has been largely described for its role in licensing Notch ligands for signal activation (10). Instead, the codependencies led quite clearly to the hypothesis that MIB1 negatively regulates one or more members of the TGF-β family.

Our examination of the TGF-β family revealed that the MIB1-dependent lines were sensitive to signaling induced by BMP but not TGF-β ligands. We validated the MIB1 dependency in individual cancer cell lines using CRISPR KO of MIB1 and conclusively demonstrated that the resulting growth inhibition was due to increased BMP signaling, as evidenced by rescue with an inhibitor specific to BMP type I receptors. Growth was not affected by inhibitors of TGF-β or Notch signaling, confirming the BMP-selective effects of MIB1 loss in this context. The BMP pathway has been well studied in development, regeneration, homoeostasis, and disease, with layers of regulation evolved to control and fine-tune its output (2, 3). Thus, our discovery not only highlights a new role for the MIB1 E3 ligase but also reveals an unexpected regulatory mechanism within the BMP signaling cascade.

To determine which step MIB1 affects in the BMP pathway, we perturbed MIB1 function using two complementary approaches: CRISPR KO, which permanently decreases MIB1 levels over several days, and dTag-induced degradation, which reversibly degrades MIB1 within 2 hours. Both methods revealed the same results, with MIB1 loss enhancing SMAD1/5/9 phosphorylation and nuclear translocation, upregulating BMP target genes, and arresting the cell cycle. RNA-seq suggested that the transcriptional effects of MIB1 loss could be primarily explained by enhanced BMP signaling. IB showed that MIB1 loss led to an increase in BMPR2 protein levels. Proteome-wide mass spectrometry identified only two proteins that showed increased expression following MIB1 loss: BMPR2 and ACVR1. Thus, this unbiased proteomic assessment not only confirmed the IB result but also narrowly focused attention on a type I and type II BMP receptor, partners in the receptor complex that binds BMP ligands (55). Together with data showing that receptor mRNA levels remained unaltered, these results lead directly to the hypothesis that MIB1 normally represses expression of BMP receptor proteins. Our model asserts that MIB1 loss depresses but does not activate BMP signaling, with signal induction still requiring a source of BMP ligand, presumably provided by the growth medium when not otherwise exogenously added.

Our studies show that MIB1 (i) represses BMPR2 signaling, (ii) downregulates BMPR2 protein in a RING-dependent manner, and (iii) physically associates with BMPR2. Although our data do not rule out the possibility of an indirect mechanism involving an intermediary adapter protein or formally establish BMPR2 as a MIB1 substrate, our results support a model in which one or more RING domains regulate BMPR2 protein abundance. Perhaps an obvious model, consistent with reports that BMP receptor turnover is regulated by ubiquitination and degradation (56, 57), would posit that MIB1 poly-ubiquitination of BMPR2 leads to BMPR2 degradation. However, long-established literature reports of MIB1 catalyzing monoubiquitination events that regulate endocytosis and trafficking–not degradation–suggest other possibilities. MIB1-catalyzed monoubiquitination of transmembrane Notch ligands induces endocytosis to generate the pulling force that activates Notch receptors (10, 18, 19, 48). Likewise, a nondegradative licensing effect of MIB1 is also observed during adenovirus nuclear translocation (16, 17). Thus, a second model holds that MIB1 monoubiquitination of BMPR2 regulates its abundance through trafficking or another nondegradative mechanism. Although elucidating the full mechanism demands further research, including whether BMPR2 is a direct MIB1 substrate, our study nevertheless physically links MIB1 to the BMP receptor complex and suggests that MIB1 regulates BMPR2 protein levels and signaling.

Our findings carry important implications for human disease. We focused on MIB1 as a strongly selective cancer dependency, and our results suggest that MIB1 inhibitors could be used to stimulate BMP signaling in tumors in which this pathway plays a critical tumor-suppressive role, including sporadic colorectal cancer, multiple myeloma, and cancers of the stomach, prostate, and esophagus (58). Given that sensitivity to MIB1 loss of function stems from increases in receptor protein levels and subsequent elevation of BMP signaling, only tumors with intact BMP pathways would be predicted to be sensitive, whereas tumors lacking core components such as SMAD4 would be predicted to be resistant. Outside of cancer, we are intrigued by our finding that MIB1 regulation of BMPR2 protein expression seems conserved across very different cell types. Decreased BMPR2 signaling is at the heart of PAH (59). Thus, we found it striking that MIB1 represses BMPR2 protein levels in primary human endothelial cells, a cell type directly relevant to PAH, highlighting another important therapeutic opportunity for MIB1 inhibition. We note that our phosphoproteomic data also provide a link between MIB1 and PAH. Specifically, phosphorylation of RASA2, a member of the RAS-GAP family, was the only protein that showed phosphorylation changes that depended on MIB1, independent of its effects on BMP signaling. Given that RASA2 is one of the genes identified in human genetic studies at the 3q22 locus that is linked to BMPR2 dysfunction in PAH (52), this raises the provocative possibility, albeit a speculative one, that RASA2 phosphorylation functions in transducing MIB1’s effect on BMPR2 protein levels.

Our results identify a new function for MIB1, outside of Notch signaling and other previously highlighted roles. Furthermore, although the BMP pathway has been thoroughly studied as fundamental to a myriad of developmental, homeostatic and disease-relevant processes, we built on publicly available genomic studies to identify MIB1 as a new negative regulator of BMP signaling. Our demonstration that loss of MIB1 RING domains correlates with increased expression of BMPR2 and elevated BMP signaling provides a rationale for developing MIB1 E3 ligase inhibitors or degraders for treating human disease, including cancers that rely on BMP tumor suppression and PAH.

## Supplementary Material

Supplementary Table S1 Key reagents and resources

Supplementary Table S2 MIB1 correlates and their roles in TGF-β family signaling.

Supplementary Table S3 Dependency scores for MIB1- and FKBP1A-dependent cell lines derived from DepMap analysis

Supplementary Table S4 Differentially expressed proteins in MIB1 KO vs. WT cells from global proteomics.

Supplementary Fig. S1 Expression of TGF-β family receptors and SMAD signaling activity in MIB1- and FKBP1A-dependent cell lines.

Supplementary Fig. S2 Validation and characterization of CRISPR-based MIB1 and FKBP1A perturbation models and associated signaling responses.

Supplementary Fig. S3 Functional assays examining the effects of MIB1 perturbation on SMAD signaling and cell growth.

Supplementary Fig. S4 Transcriptomic analysis of gene expression changes following modulation of BMP signaling.

Supplementary Fig. S5 Proteomic workflow and phosphorylation analysis under different BMP signaling conditions.

## Data Availability

RNA-seq data have been deposited in the NCBI Gene Expression Omnibus (GEO) under accession number GSE275450. Proteomics data have been deposited in the MassIVE repository under dataset identifier MSV000095215 (https://doi.org/10.25345/C5DZ03C9D). Data from the DepMap project at the Broad Institute were obtained from the DepMap portal (https://depmap.org/portal). The version of the dataset used in this study is described in the DepMap datamining of the “Materials and Methods” section. Other data generated in this study are available upon request to the corresponding author.
