# Supplementary material for: E3 Ubiquitin Ligase Mind Bomb 1 is a Novel Negative Regulator of Tumor-Suppressive BMP Signaling
Source: Cancer Res Commun. 2026 Aug 17;6(8):1943–62. doi: 10.1158/2767-9764.CRC-25-0760 (PMC13489675; doi:10.1158/2767-9764.CRC-25-0760)
Supplement: Supplementary file 1 [file crc-25-0760_supplementary_table_s1_suppst1.pdf]

Supplementary Table S1. Key resources used in this study.

Reagent type (species) or resource	Designation	Source or reference	Identifiers	Additional information
cell line (Homo sapiens)	BT549	Genentech	RRID:CVCL_1092	Female
cell line (Homo sapiens)	CAMA1	Genentech	RRID:CVCL_1115	Female
cell line (Homo sapiens)	HCC515	Genentech	RRID:CVCL_5136	Male
cell line (Homo sapiens)	HCC1187	Genentech	RRID:CVCL_1247	Female
cell line (Homo sapiens)	HCC1419	Genentech	RRID:CVCL_1251	Female
cell line (Homo sapiens)	NCIH28	Genentech	RRID:CVCL_1555	Male
cell line (Homo sapiens)	NCIH292	Genentech	RRID:CVCL_0455	Female
cell line (Homo sapiens)	NCIH838	Genentech	RRID:CVCL_1594	Male
cell line (Homo sapiens)	HEK293T	Genentech	RRID:CVCL_0V13	—
cell line (Homo sapiens)	HUVEC	Lonza	C2519A	Mixed sex; pooled donor; passages 3–9
transfected construct (Homo sapiens)	pBIND-Cas9-sgRNA-MIB1	This paper	—	Dox-inducible KO system
transfected construct (Homo sapiens)	pBIND-Cas9-sgRNA-FKBP1A	This paper	—	Dox-inducible KO system
sequence-based reagent	MIB1 sgRNA #1	This paper	—	5′-TTGGCGCTCGGGTAGTGCG-3′
sequence-based reagent	MIB1 sgRNA #2	This paper	—	5′-GCCAACTACCGCTGCTCCG-3′
sequence-based reagent	FKBP1A sgRNA	This paper	—	5′-CACTACTCACCGTCTCCT-3′
recombinant DNA reagent	MIB1 wt cDNA	This paper	—	Cas9-resistant, EF1α-IRES-mNeonGreen
recombinant DNA reagent	MIB1 RING Δ1-3	This paper	—	aa 819–1006 deletion
recombinant DNA reagent	dTAG-MIB1	This paper	—	FKBP12F36V tagged, EF1α-IRES-mNeonGreen
recombinant DNA reagent	mNeonGreen control vector	This paper	—	Lentiviral or piggyBac
peptide, recombinant protein	TGF-β2	Genentech	PUR171061	Purified from CHO cells
peptide, recombinant protein	BMP2	Gibco	PHC7145	—

peptide, recombinant protein	BMP4	Gibco	PHC9534	—
peptide, recombinant protein	BMP7	Gibco	PHC9544	—
peptide, recombinant protein	BMP9	Novus	3209-BP-010/CF	—
peptide, recombinant protein	TGF-β1	Gibco	PHG9214	—
peptide, recombinant protein	TGF-β3	PeproTech	AF-100-36E	—
chemical compound, drug	Compound-E	Stem Cell Technologies	73952	—
chemical compound, drug	LDN-193189	Tocris	6053	—
chemical compound, drug	SB-431542	Tocris	1614	—
chemical compound, drug	DAPI stain	Invitrogen	D1306	0.2 μg/ml
commercial assay, kit	CellTiter-Glo	Promega	G7571	Cell viability
commercial assay, kit	Click-it EdU Proliferation Kit	Invitrogen	C10419	Cell cycle assay
commercial assay, kit	RNeasy Plus Kit	Qiagen	74134	—
commercial assay, kit	RNeasy Micro Kit	Qiagen	74004	—
commercial assay, kit	RNA HS Assay Kit	Thermo Fisher	Q32852	—
commercial assay, kit	dsDNA HS Assay Kit	Thermo Fisher	Q32851	—
commercial assay, kit	Taqman RNA-to-Ct Kit	Thermo Fisher	4392938	RT-qPCR
sequence-based reagent	MIB1 Taqman probe	Thermo Fisher	4351372; Hs01075899_m1	—
sequence-based reagent	BMPR2 Taqman probe	Thermo Fisher	4331182; Hs00176148_m1	—
sequence-based reagent	GAPDH Taqman probe	Thermo Fisher	4331182; Hs02786624_g1	—
commercial assay, kit	Nextera XT DNA Kit	Illumina	FC-131-1096	—
commercial assay, kit	Truseq Stranded mRNA Kit	Illumina	20020594	—
commercial assay, kit	Smart-Seq V4 Ultra Low Kit	Takara	634888	—
commercial assay, kit	TMTPro16-plex	ThermoFisher	—	Isobaric peptide labeling
commercial	Sep-Pak C18	Waters	—	Peptide cleanup

assay, kit	column			
commercial assay, kit	pY PTMScan Kit	Cell Signaling	8803	pY enrichment
commercial assay, kit	Cell Surface Biotinylation Kit	Thermo Fisher	A44390	—
antibody	anti-MIB1	Abcam	ab124929, RRID:AB_11127834	immunoblot1:1000
antibody	anti-MIB1	Thermo Fisher	MA5-31797	immunoblot1:500
antibody	anti-BMPR2	Abcam	ab96826, RRID:AB_10679307	immunoblot 1:1000; immunofluorescence 1:200
antibody	anti-pSMAD1/5/9	Cell Signaling	#13820, RRID:AB_2493181	immunoblot 1:1000; immunofluorescence 1:400
antibody	anti-pSMAD1/5	Cell Signaling	#9516, RRID:AB_491015	immunoblot 1:1000
antibody	anti-pSMAD2/3	Cell Signaling	#9510, RRID:AB_2193178	immunoblot 1:1000; immunofluorescence 1:400
antibody	anti-β-Actin	Cell Signaling	#3700, RRID:AB_2242334	immunoblot 1:2000
antibody	anti-Cas9	Cell Signaling	#14697, RRID:AB_2750916	immunofluorescence 1:500
antibody	anti-p21	Cell Signaling	#2947, RRID:AB_823586	immunoblot 1:1000; immunofluorescence 1:200
antibody	anti-SMAD1	Cell Signaling	#6944, RRID:AB_10858882	immunoblot 1:1000
antibody	anti-SMAD1	Abcam	ab53745	immunoblot 1:100
antibody	Alexa Fluor-conjugated secondary	Invitrogen	—	immunofluorescence 1:200
antibody	HRP-conjugated secondary	Jackson ImmunoResearch	—	immunoblot 1:5000
antibody	IRDye-conjugated secondary	LI-COR	—	immunoblot 1:10,000
software, algorithm	Google Colab	Google	RRID:SCR_008394	Python 3 scripting
software, algorithm	HTSeqGenie	Bioconductor	RRID:SCR_005514	Read counting
software, algorithm	voom (limma R package)	Bioconductor	RRID:SCR_010943	Normalization
software, algorithm	clusterProfiler	Bioconductor	RRID:SCR_016884	GSEA
software, algorithm	GSEA Desktop	Broad Institute	RRID:SCR_003199	GSEA software
software, algorithm	MSstatsTMT	Bioconductor	DOI:10.18129/B9.bioc.MSstatsTMT	Quantification
software, algorithm	MSstatsPTM	Bioconductor	DOI:10.18129/B9.bioc.MSstatsPTM	PTM normalization
software, algorithm	Mojave	This paper	—	TMT-MS3 quantification

software, algorithm	Mascot	Matrix Science	RRID:SCR_000307	Database search
software, algorithm	CellProfiler	Broad Institute	RRID:SCR_007358	Image analysis
software, algorithm	GraphPad Prism	GraphPad	RRID:SCR_002798	Statistics
software, algorithm	FlowJo	BD Biosciences	RRID:SCR_008520	Flow cytometry analysis
software, algorithm	Opera Phenix + Columbus	PerkinElmer	—	immunofluorescence acquisition/analysis
