# Supplementary material for: E3 Ubiquitin Ligase Mind Bomb 1 is a Novel Negative Regulator of Tumor-Suppressive BMP Signaling
Source: Cancer Res Commun. 2026 Aug 17;6(8):1943–62. doi: 10.1158/2767-9764.CRC-25-0760 (PMC13489675; doi:10.1158/2767-9764.CRC-25-0760)
Supplement: Supplementary file 2 [file crc-25-0760_supplementary_table_s2_suppst2.pdf]

Supplementary Table S2. MIB1 correlates and their roles in TGF-β family signaling.

Official Symbol	Official Full Name	Also Known As	Pathway Function	Regulatory Effect	Refs
FKBP1A	FKBP prolyl isomerase 1A	FKBP12	Receptor-associated modulator	Negative (prevents ligand-independent activation)	27-29
BAMBI	BMP and activin membrane-bound inhibitor	NMA	Pseudoreceptor	Negative (inhibits receptor complex formation)	30
ID3	Inhibitor of DNA binding 3, HLH protein		Transcriptional effector	Context-dependent mediator	31
SMAD7	SMAD family member 7		Inhibitory SMAD	Negative (feedback inhibitor)	32, 34
SMAD6	SMAD family member 6		Inhibitory SMAD	Negative (primarily BMP-specific)	35, 36
IPO8	Importin 8	RANBP8	Nuclear import factor	Positive (transports SMADs to nucleus)	8, 37
RGMB	RGM domain family member B	DRAGON	Co-receptor	Positive (enhances BMP signaling)	26
ENAH	Enabled homolog (Drosophila)	MENA	Cytoskeletal-associated effector	Context-dependent (may mediate signaling outputs)	38
ID1	Inhibitor of DNA binding 1		Transcriptional effector	Context-dependent mediator	39
LEMD3	LEM domain containing 3	MAN1	Inner nuclear membrane protein	Negative (binds SMADs to repress transcription)	40, 41

Genes listed here were identified as top MIB1 correlates by DepMap analysis (Fig. 1C). Their known or proposed roles in TGF-β or BMP signaling are summarized based on literature review, with references provided.
