# Supplementary material for: E3 Ubiquitin Ligase Mind Bomb 1 is a Novel Negative Regulator of Tumor-Suppressive BMP Signaling
Source: Cancer Res Commun. 2026 Aug 17;6(8):1943–62. doi: 10.1158/2767-9764.CRC-25-0760 (PMC13489675; doi:10.1158/2767-9764.CRC-25-0760)
Supplement: Supplementary file 3 [file crc-25-0760_supplementary_table_s3_suppst3.pdf]

Supplementary Table S3. Dependency scores for MIB1- and FKBP1A-dependent cell lines.

	DepMap_ID	Cell line	Lineage	MIB1 dependency score	FKBP1A dependency score
1	ACH-000648	NCIH28 *	Pleura	-1.59	-0.58
2	ACH-001096	JR	Soft Tissue	-1.55	-0.81
3	ACH-001377	PACADD137	Pancreas	-1.48	-0.71
4	ACH-001099	KD	CNS/Brain	-1.45	-1.16
5	ACH-000804	NB1 *	Peripheral Nervous System	-1.45	-0.56
6	ACH-000416	NCIH838 *	Lung	-1.40	-0.73
7	ACH-002239	H3118	Head and Neck	-1.38	-0.68
8	ACH-000441	SH4	Skin	-1.35	-0.98
9	ACH-000559	NCIH1836	Lung	-1.33	-1.39
10	ACH-000872	HCC515*	Lung	-1.16	-0.67
11	ACH-000277	HCC1419*	Breast	-1.14	-0.67
12	ACH-000280	SNU840	Ovary/Fallopian Tube	-1.10	-1.19
13	ACH-001610	NP5	CNS/Brain	-1.08	-0.84
14	ACH-001616	OCILY18	Lymphoid	-1.06	-1.59
15	ACH-001020	BT16	CNS/Brain	-1.04	-0.57
16	ACH-001354	LAN2	Peripheral Nervous System	-1.03	-0.68
17	ACH-001163	CCLFPEDS0001T	Kidney	-1.02	-0.53
18	ACH-001031	CHLA266	CNS/Brain	-1.00	-0.51
19	ACH-000161	CORL105	Lung	-0.98	-0.77
20	ACH-001993	NALM16	Lymphoid	-0.97	-0.94
21	ACH-000581	SNU16	Esophagus/Stomach	-0.96	-0.85
22	ACH-000209	SNU1079	Biliary Tract	-0.90	-0.53
23	ACH-000995	JURKAT	Lymphoid	-0.90	-0.77
24	ACH-000533	NCIH2004RT	Kidney	-0.90	-0.54
25	ACH-000734	JHH5	Liver	-0.88	-0.66
26	ACH-002024	ECC4	Bowel	-0.88	-0.54
27	ACH-000480	HUH7	Liver	-0.86	-0.64
28	ACH-001500	FLO1	Esophagus/Stomach	-0.84	-0.61
29	ACH-000426	KMS20	Lymphoid	-0.83	-0.59
30	ACH-000407	SNU685	Uterus	-0.81	-0.78
31	ACH-000288	BT549 *	Breast	-0.80	-0.50
32	ACH-000720	TCCSUP	Bladder/Urinary Tract	-0.79	-0.56
33	ACH-001532	JMURTK2	Kidney	-0.79	-0.57
34	ACH-000111	HCC1187 *	Breast	-0.78	-0.60
35	ACH-000094	HPAFII	Pancreas	-0.76	-0.56

36	ACH-000503	BICR16	Head and Neck	-0.72	-0.50
37	ACH-000512	INA6	Lymphoid	-0.68	-0.68
38	ACH-000460	SNU8	Ovary/Fallopian Tube	-0.67	-0.99
39	ACH-000783	CAMA1 *	Breast	-0.66	-0.60
40	ACH-001416	UMUC9	Bladder/Urinary Tract	-0.64	-0.57
41	ACH-000422	SNU475	Liver	-0.61	-0.59
42	ACH-001075	NCIH292 *	Lung	-0.60	-0.53
43	ACH-000095	D341Med	CNS/Brain	-0.59	-0.54
44	ACH-001737	CTV1DM	Lymphoid	-0.59	-0.52
45	ACH-000810	SKMEL30	Skin	-0.56	-0.75
46	ACH-000805	COLO679	Skin	-0.54	-0.73
47	ACH-000597	TTC709	CNS/Brain	-0.53	-0.56
48	ACH-000556	SIHA	Cervix	-0.53	-0.55
49	ACH-000782	SEM	Lymphoid	-0.52	-0.74

This table supplements Fig. 2A and reports Chronos gene dependency scores for MIB1 and FKBP1A across 49 cancer cell lines from the DepMap Public 22Q4 CRISPR dataset. Cell lines used experimentally in this study are indicated with an asterisk (*).
