# Supplementary material for: E3 Ubiquitin Ligase Mind Bomb 1 is a Novel Negative Regulator of Tumor-Suppressive BMP Signaling
Source: Cancer Res Commun. 2026 Aug 17;6(8):1943–62. doi: 10.1158/2767-9764.CRC-25-0760 (PMC13489675; doi:10.1158/2767-9764.CRC-25-0760)
Supplement: Supplementary file 4 [file crc-25-0760_supplementary_table_s4_suppst4.pdf]

Supplementary Table S4. Differentially expressed proteins in MIB1 knockout vs. wild-type cells from global proteomics.

Gene Name	Protein Name	UniProt Identifier	Fold change (log2)	-log10(p-value)
MIB1	E3 ubiquitin-protein ligase MIB1	MIB1_HUMAN|Q86YT6	-2.35	9.22
BMPR2	Bone morphogenetic protein receptor type-2	BMPR2_HUMAN|Q13873	1.17	8.22
CRYBG2	crystallin beta-gamma domain containing 2	E7ET48_HUMAN|E7ET48	-1.28	7.93
CDKN2C	Cyclin-dependent kinase 4 inhibitor C	CDN2C_HUMAN|P42773	0.88	7.63
CRYBG2	crystallin beta-gamma domain containing 2	E9PIR9_HUMAN|E9PIR9	-1.34	7.07
SAA1	Serum amyloid A-1 protein	SAA1_HUMAN|P0DJI8	-1.33	5.60
S100A9	Protein S100-A9	S10A9_HUMAN|P06702	-1.82	5.41
TM7SF2	Delta(14)-sterol reductase	ERG24_HUMAN|O76062	0.60	4.51
RHOB	Rho-related GTP-binding protein RhoB	RHOB_HUMAN|P62745	-0.60	3.96
SLC7A2	Cationic amino acid transporter 2	CTR2_HUMAN|P52569	-0.64	3.56
CPTP	Ceramide-1-phosphate transfer protein	CPTP_HUMAN|Q5TA50	-0.59	3.51
PRC1	Protein regulator of cytokinesis 1 (isoform 2)	PRC1_HUMAN|O43663-2	-0.83	3.41
CRIP2	Cysteine-rich protein 2	CRIP2_HUMAN|P52943	0.60	3.18
SLPI	Antileukoproteinase	SLPI_HUMAN|P03973	-0.59	2.73
VTN	Vitronectin	VTNC_HUMAN|P04004	-0.73	2.42
ACVR1	Activin receptor type-1	ACVR1_HUMAN|Q04771	1.20	2.19
NMES1	Normal mucosa of esophagus-specific gene 1 protein	NMES1_HUMAN|Q9C002	-0.60	2.07
KIAA0101	PCNA-associated factor	PAF15_HUMAN|Q15004	-0.69	1.89
IL6	Interleukin-6	IL6_HUMAN|P05231	-1.01	1.46

This table supplements Fig. 7A and reports proteins from the global proteomics experiment that showed ≥1.5-fold change in expression between MIB1 knockout and wild-type cells. p-values <0.05 are included.
